# Nickel sulfide and phosphide electrocatalysts for hydrogen evolution reaction: challenges and future perspectives

**DOI:** 10.1039/d2ra04897c

**Published:** 2022-10-17

**Authors:** Ali Shahroudi, Mahsa Esfandiari, Sajjad Habibzadeh

**Affiliations:** Surface Reaction and Advanced Energy Materials Laboratory, Chemical Engineering Department, Amirkabir University of Technology (Tehran Polytechnic) Tehran Iran sajjad.habibzadeh@mail.mcgill.ca

## Abstract

The search for environmentally friendly and sustainable energy sources has become necessary to alleviate the issues associated with the consumption of fossil fuel such as air pollution and global warming. Furthermore, this is significant considering the exhaustible resources and burgeoning energy demand globally. In this regard, hydrogen, a clean fuel with high energy density, is considered a reliable alternative energy source. The hydrogen evolution reaction (HER) is one of the most promising methods to produce green hydrogen from water on a large scale. However, the HER needs effective electrocatalysts to address the concerns of energy consumption; thus, finding active materials has recently been the main focus of researchers. Among the various electrocatalysts, nickel sulfides and phosphides and their derivatives with low cost, high abundance, and relatively straightforward preparation have shown high HER activity. In this review, we compare the diverse methods in the synthesis of nickel sulfides and phosphides together with effective synthesis parameters. Also, the optimum conditions for the preparation of the desired active materials and their properties are provided. Then, the performance of nickel sulfide and phosphide electrocatalysts in the HER is addressed. The HER activity of the various crystalline phases is compared, and their most active crystalline phases are introduced. Finally, the present challenges and perspectives for future HER electrocatalysts are presented.

## Abstract 2

A comprehensive overview of nickel sulfide and phosphide electrocatalysts in the HER.

## Graphical abstract

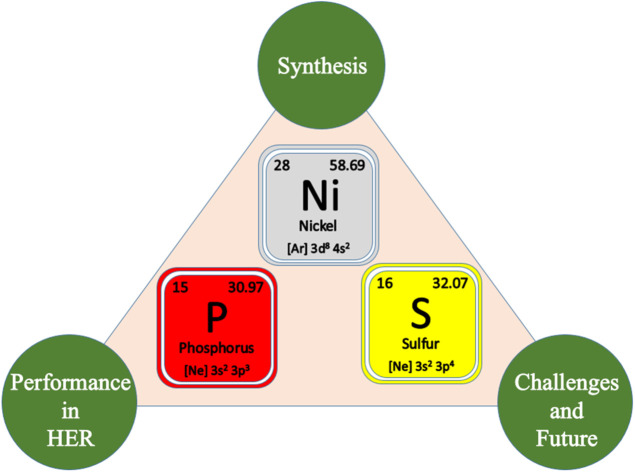

## 1. Introduction

Nowadays, due to the burgeoning world energy demand and exhaustible fossil fuel resources, renewable energies have received much attention.^1,2^ In the last two centuries, although fossil fuels as key energy carriers have played a significant role in industrial development, they are non-renewable sources and their formation through natural processes takes millions of years. Also, there are many environmental concerns including air pollution and global warming caused by combustion.^3,4^ Thus, finding sustainable energy sources to replace fossil fuels is vital.

**Fig. 1:**
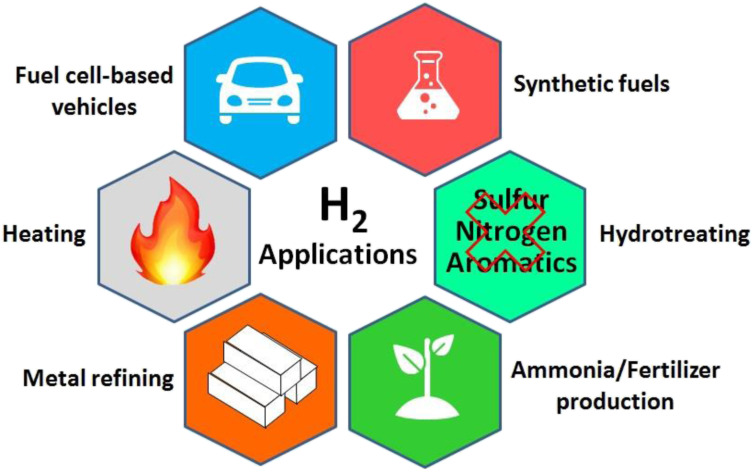
Graphical representation of the applications of hydrogen.

Renewable energies such as solar and wind energy are potential substitutes for fossil fuels. However, their intrinsic fluctuation has led to their low energy efficiency and has limited their usage. Accordingly, storing the energy from these types of sources in the chemical bonds of molecules is a viable solution.^5^ In this regard, one of the best energy storage molecules is hydrogen. The high energy density (about three times greater than standard fuels such as natural gas and gasoline) and clean hydrogen combustion demonstrate that it is as a considerable alternative to fossil fuels.^6,7^ In addition, currently, hydrogen plays a crucial role in many applications, making it and its production methods critical. Fig. 1 shows a graphical representation of the applications of hydrogen.

Hydrogen is mainly produced by natural gas and oil reforming or coal gasification. However, these processes consume a great amount of fossil fuels, emit CO_2_, and have low energy efficiency due to severe operational conditions. Also, the presence of carbon residue and sulfur contaminants leads to the production of hydrogen with low purity, which can easily poison sensitive catalysts.^8^ Thus, it is necessary to develop suitable strategies for the efficient and clean production of pure hydrogen.

The hydrogen evolution reaction (HER), the cathodic half-reaction of water electrolysis, is a clean and efficient alternative energy source, which produces highly pure hydrogen from water or aqueous solutions at ambient pressure and temperature without undesirable emissions. Similar to many other electrochemical processes, the HER requires electrocatalysts with high electrocatalytic activity, long-term stability, and low price for sustainable hydrogen production to overcome the energy barriers. Accordingly, Pt-group metals are the best electrocatalysts for the HER in terms of activity. However, their high cost and scarcity limit their use.^9,10^ Thus, significant research efforts have been devoted to finding effective non-noble metal electrocatalysts. In this regard, nickel sulfides, phosphides and their derivatives with low cost, high abundance, and relatively easy preparation have shown high activity toward the HER and are known as promising materials for the efficient electrocatalysis of the HER. However, these materials have not been studied in detail. Hence, in this review article, we provide a complete overview of these materials.

This review introduces the fundamentals of the HER and approaches for evaluating electrocatalysts in detail. Then, the different methods for the synthesis of nickel sulfides and phosphides, including the electrodeposition technique, solvothermal route, thermal decomposition, and vapor–solid reaction, are investigated and compared, their effective parameters and how they affect the properties of the synthesized materials are comprehensively discussed, and the best parameters for obtaining the desired materials and properties are provided. Then, the performance of nickel sulfide and phosphide electrocatalysts in the HER is studied, the HER activity of their different crystalline phases is compared, and their most active crystalline phase is introduced. Finally, the present challenges and perspectives for future works are presented.

## 2. Fundamentals of the HER

The overall water splitting reaction is as follows:1
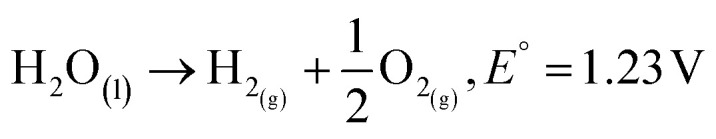


The HER is the cathodic half-reaction of the overall water splitting reaction, and its mechanism consists of two main steps. The first step is the electrochemical hydrogen ion adsorption reaction, which is known as the Volmer step (discharge reaction). In this step, a proton or water molecule reacts with an electron in acidic or alkaline media to produce an adsorbed hydrogen atom (H*) on the electrode surface (see reactions (^2^) and (^3^)).

Acidic medium:

2 H^+^ + e^−^ → H*

Alkaline medium:

3 H_2_O + e^−^ → H* + OH^−^

In the second step, molecular hydrogen is formed, which can occur through a chemical desorption reaction (Tafel reaction) or electrochemical desorption reaction (Heyrovsky reaction). In the Heyrovsky reaction, a hydrogen ion (H^+^) or water molecule in the electrolyte combines with an adsorbed hydrogen (H*) on the electrode and an electron to produce one hydrogen molecule.

Acidic medium:

4 H* + H^+^ + e^−^ → H_2_

Alkaline medium

5 H* + H_2_O + e^−^ → H_2_ + OH^−^

Two adsorbed hydrogens (H*) combine to form molecular hydrogen in the Tafel reaction.

6 H* + H* → H_2_

Thus, in general, the HER has two mechanisms, *i.e.*, Volmer–Heyrovsky and Volmer–Tafel. The classification of the reactions based on acidic and alkaline media shows which reaction is dominant but does not mean that only the mentioned reaction proceeds in a particular medium. Also, hydrogen is produced in an electrolyte through both Volmer–Heyrovsky and Volmer–Tafel mechanisms, not just one of them. The Tafel plot can be employed to determine the dominant mechanism and the rate-determining step, which will be discussed in Section 3.2.

## 3. Evaluation criteria of HER electrocatalysts

### 3.1. Overpotential

In the equilibrium state, the net current density is zero, and to perform an electrolytic reaction at current densities greater than zero, energy more than the equilibrium electromotive force have to be applied to the system. This extra energy is used to overcome several energy barriers and resistances such as electron transfer resistance and mass transfer resistance. The difference between the applied and equilibrium electromotive forces is the overpotential (*η*).

7 *η* = *E*_applied_ − *E*_equilibrium_

The overpotential is one of the main evaluation parameters of electrocatalysts. The lower the overpotential an electrocatalyst needs to deliver a specific current density, the lower the amount of energy is consumed, and thus the electrocatalyst shows a better performance. Commonly, electrocatalysts are evaluated and compared based on the required overpotentials at current densities of 1, 10 and 100 mA cm^−2^. The overpotential at 1 mA cm^−2^, which is mainly known as the onset potential, shows the intrinsic activity of electrocatalysts for triggering an electrochemical reaction and significantly affects the overall performance. The current density of 10 mA cm^−2^ is the current density on which photovoltaic cells usually operate, and the current density of 100 mA cm^−2^ has been selected as a criterion to show the performance of electrocatalysts on an industrial scale. These values are measured by linear sweep voltammetry (LSV). It should be noted that usually, the ohmic loss due to the resistance of the electrolyte (*R*_s_) is compensated to investigate only the performance of electrocatalysts (*E*_LSV_ = *E*_measured_ − *iR*_s_).

In the HER field, the electric potential is often reported relative to the reversible hydrogen electrode (RHE). However, reference electrodes such as Ag/AgCl and saturated calomel electrode (SCE) are commonly used in practice. The following equations can be employed to convert the potential measured by these two reference electrodes with saturated electrolyte at 25 °C:^11^

8 *E*_RHE_ (V) = *E*_Ag/Agcl_ (V) + 0.199 + 0.059 × pH

9 *E*_RHE_ (V) = *E*_SCE_ (V) + 0.244 + 0.059 × pH

In the case of the use of these two reference electrodes at different temperatures and concentrations, the above-mentioned equations must be corrected accordingly.

### 3.2. Tafel plot

According to the previous section, the overpotential is dependent on the current density. The relationship between the overpotential and current density, where electron transfer controls the electrochemical reaction (most cases), can be explained by the Butler–Volmer equation, as follows:10
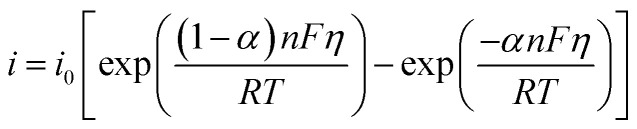
where *η*, *i*, *i*_0_, *α*, *n*, *F*, *R*, and *T* are the overpotential, current density, exchange current density, transfer coefficient, number of electrons exchanged, Faraday constant, gas constant, and temperature, respectively. Eqn (10) can be simplified based on the cathodic or anodic nature of the reaction and the range of the overpotential. In the HER, which is a cathodic reaction, at high overpotentials, eqn (10) can be simplified as follows:11
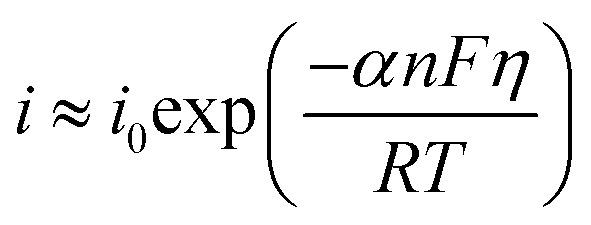


Extraction of *η* and converting the logarithm base from *e* to 10 give the following equation:12
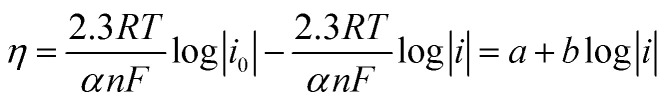


As is evident, the overpotential against the logarithm of current density is a straight line with the slope of 
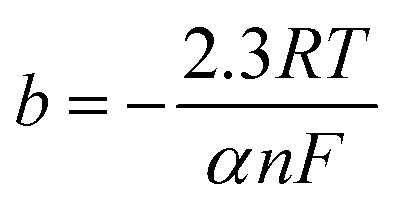
 and intercept of 
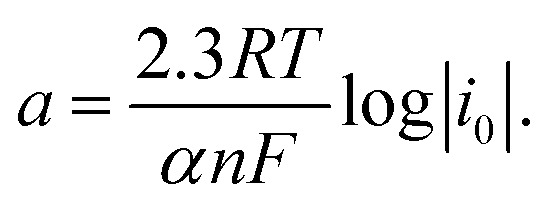
 This linear equation and slope (*b*) are called the Tafel equation and Tafel slope, respectively, which were proposed for the first time in 1905 by Julius Tafel.

The values of *a* and *b* are usually obtained from LSV. Plotting the overpotential against the logarithm of current density gives a curve with a linear part, which can be interpolated with a line equation, and *a* and *b* are obtained from the intercept and slope of this line, respectively. The exchange current density can be determined from *a* according to its definition. It is worth mentioning that the Tafel slope value can also be obtained using EIS, which will be explained in Section 3.3.

The unit of Tafel slope is mV dec^−1^, which indicates how much overpotential has to be applied to increase the current density by 10-fold. Thus, the lower the Tafel slope of an electrocatalyst, the better its performance. The exchange current density is the current density at the equilibrium state (zero overpotential), which indicates the intrinsic activity of electrocatalysts. Thus, the higher the exchange current density of an electrocatalyst, the better its electrocatalytic performance.

Besides the above-mentioned application of the Tafel slope for the evaluation of electrocatalysts, it also can be used to determine the dominant HER mechanism and the rate-determining step (RDS) of the HER. The Tafel slopes of the Volmer, Heyrovsky, and Tafel reactions at 25 °C and assuming *α* = 0.5 are 118, 39 and 30 mV dec^−1^, respectively. Based on these values, the RDS of the HER can be specified. For example, if the Tafel slope of an electrocatalyst is more than 118 mV dec^−1^, the Volmer reaction (electrochemical adsorption) controls the HER or if the Tafel slope is between 118 and 39 mV dec^−1^, the HER mechanism is the Volmer–Heyrovsky, and the HER proceeds through a relatively fast Volmer reaction and a controlling Heyrovsky reaction.

### 3.3. Conductivity/charge transfer resistance

Conductivity or charge transfer resistance is one of the essential characteristics of electrocatalysts. Specifically, the higher the conductivity of an electrocatalyst, the less energy will be wasted during charge transfer. One of the most accurate and advanced techniques for measuring and comparing the charge transfer resistance of electrocatalysts is electrochemical impedance spectroscopy (EIS) (impedance in alternating current (AC) systems is equivalent to the resistance in direct current (DC) systems). In this technique, the impedance of electrocatalysts is measured by applying an AC voltage with a constant amplitude and frequency usually in the range of 100 kHz to 100 mHz. The use of an alternating current enables the effects of resistances caused by redox reactions to be considered or eliminated by adjusting the frequency. One of the outputs of EIS is the Nyquist plot, which depicts the imaginary parts of impedances against the real parts. In the case of electrocatalysts, which usually have both capacitive and resistive behavior, the Nyquist plot is approximately a semi-circle. The beginning of the plot (semi-circle) at high frequencies shows the resistance of the electrolyte. This is because redox reactions cannot proceed at high frequencies due to the quick change of the anode and cathode, and only the movement and the conductivity of ions are monitored. The end of the plot (semi-circle) at low frequencies (close to zero) in which the current is somehow direct shows all the resistances of the system, including the charge transfer resistance of the electrolyte and electrocatalyst. Thus, the diameter of the semi-circle indicates the charge transfer resistance of the electrocatalyst (note that in general, fitting techniques should be applied to extract the resistance values). To compare different electrocatalysts, the smaller the diameter of the EIS semi-circle of an electrocatalyst, the smaller its charge transfer resistance, and in terms of conductivity, it is the best electrocatalyst. Note that Nyquist plots and EIS are only addressed practically here, while their fundamentals are more than explained. Thus, it is suggested that EIS be studied in detail in the relevant sources.

In addition to evaluating conductivity, a newly developed application of EIS is the calculation of the Tafel slope.^12^ To do this, EIS is performed at different amplitudes, and in each case, the charge transfer resistance (*R*_ct_) is extracted. Then, the amplitude is plotted *versus*
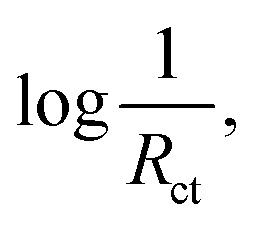
 which gives a straight line, with its slope equal to the Tafel slope of the electrocatalyst. The Tafel slope calculated by this method is more accurate than that calculated using LSV data because only the charge transfer resistance of the electrocatalyst is included, and the other resistances of the system, such as the resistance of the electrolyte, are not considered.

### 3.4. Stability and durability

Stability/durability is one of the most important properties of electrocatalysts, especially in industrial applications. This is because with the use of more stable and durable electrocatalysts, maintenance costs are lowered, and thus processes become more cost-effective. The stability of electrocatalysts is usually evaluated using two electrolysis approaches, *i.e.*, galvanostatic polarization (chronopotentiometry) and potentiostatic polarization (chronoamperometry). In chronoamperometry/chronopotentiometry, the current density/overpotential is tracked against time at a constant overpotential/current density. After a period of time, if the change in potential or current density is negligible, the electrocatalyst is considered stable. It is worth mentioning that the currently used electrocatalysts in industrial water electrolyzers have shown a stable operation for as long as 80 000 h.^13^ The durability of electrocatalysts can be assessed by continuous cyclic voltammetry (CV) tests. In this method, the performance of the electrocatalyst is evaluated by LSV plots before and after applying several continuous CV or LSV tests. The smaller the difference between the LSV curves of an electrocatalyst, the more durable it is.

### 3.5. Electrochemical active surface area

Three types of surfaces can be considered for an electrocatalyst, as follows: (1) geometric surface, (2) physical specific surface area (SSA) and (3) electrochemical active surface area (ECSA). The geometric surface is the area that can be seen visually and measured using simple tools such as a ruler. This surface usually is used to report the current density relative to the surface area. The SSA is the total surface area of a material exposed per mass unit, which is measured by gas adsorption methods. However, the whole SSA is not active for reactions, and an electrocatalyst with a high SSA does not necessarily have high electrocatalytic activity. Thus, the surface area that is active for a reaction becomes important in the field of electrochemical reactions, which is called the electrochemical active surface area (ECSA). The higher the ECSA per unit of mass of an electrocatalyst, the more active sites it provides for the desired reaction, resulting in a better electrocatalytic performance. In the HER field, usually a CV test is used to calculate the ECSA. In this case, initially, the open circuit potential (OCP), which is the working potential of the electrode without applying any load to the system, is measured. The CV test is conducted at various scan rates in a potential range around the OCP. This potential range is selected because the current density is almost non-faradaic, and the electrical energy is consumed to store charges on the surface of the electrode. Then, the current density is plotted *versus* the scan rate, which gives a line with a slope equal to the electrochemical double-layer capacitance (*C*_dl_) of the electrocatalyst. The selected current densities must be non-faradaic (charging) and almost in the plateau part of the CV curves selected at the OCP. To consider both the anodic and cathodic paths in CV, the difference between the cathodic and anodic current density is commonly plotted against scan rate. Accordingly, it must be noted that the *C*_dl_ is equal to half of the slope. The *C*_dl_ is directly proportional to the ECSA. Thus, the higher the *C*_dl_, the higher the ECSA. Based on the type of materials and the capacitance per unit of area (*C*_s_), the ECSA can be calculated according to the following equation:

13 ECSA = *C*_dl_/*C*_s_

### 3.6. Turnover frequency

The electrocatalytic performance of electrocatalysts and their intrinsic activity is a function of their mass loading and SSA. Different electrocatalysts have different loadings and SSAs, making it difficult to compare their intrinsic activity. For example, an electrocatalyst with lower intrinsic activity but higher mass loading or SSA may perform better than an electrocatalyst with higher intrinsic activity but lower mass loading or SSA. Thus, comparing the intrinsic activity of electrocatalysts is challenging, and defining a parameter that can indicate the intrinsic activity of materials regardless of the mass loading and SSA is of great importance. The turnover frequency (TOF) is obtained by normalizing the amount of evolved hydrogen in relation to the number of active sites, which can indicate the intrinsic activity of electrocatalysts and used to compare different electrocatalysts in a meaningful manner. In the HER field, the TOF is defined as the number of hydrogen molecules produced per number of active sites in one second. According to this definition, the higher the TOF of an electrocatalyst, the higher its intrinsic activity. Because the number of produced H_2_ per unit time depends on the current density and overpotential, the TOF of different electrocatalysts is compared at the same overpotentials. At the same overpotentials, an electrocatalyst with a higher TOF has higher intrinsic activity toward the HER. It should be noted that the number of active sites is calculated using the ECSA based on some assumptions that make calculating the exact number of active sites inaccessible. Nevertheless, the TOF can be considered an acceptable parameter for comparing the intrinsic activity of different electrocatalysts.

### 3.7. Hydrogen adsorption

Hydrogen adsorption is one of the most important aspects of HER electrocatalysis. Materials with moderate (neither too low nor too high) hydrogen adsorption ability show better performances in the HER than materials with too low or too high hydrogen adsorption ability. In detail, according to the Sabatier principle,^14^ the closer to zero the Gibbs free energy of hydrogen adsorption (Δ*G*_H_), the better the HER performance. This is because although materials with high hydrogen adsorption ability (Δ*G*_H_ < 0) can adsorb hydrogen species such as H^+^ or H_2_O very well, they cannot properly release hydrogen in the desorption steps (Heyrovsky or Tafel reaction), thus limiting the reaction rate. Alternatively, materials with low hydrogen adsorption ability (Δ*G*_H_ > 0) will face the problem from the first step (Volmer reaction) because they cannot adsorb reactants of the HER well. Therefore, the best electrocatalysts in terms of hydrogen adsorption are those that have a closer to zero Δ*G*_H_. In addition to being used as a criterion for hydrogen adsorption ability, Δ*G*_H_ is also used in combination with the exchange current density to provide a good view for comparing the HER activity of different materials. For this purpose, the exchange current densities of materials are depicted *versus* their Δ*G*_H_, which gives a semi theoretical-semi experimental volcano-type plot. In the volcano plot, the best materials are at the top of the plot, which are Pt-group metals. Thus, the closer to the top of the volcano plot the location of an electrocatalyst, the better it is.

## 4. Synthesis methods

### 4.1. Electrodeposition technique

Electrodeposition is an easy synthesis method with mild conditions in which both synthesis and deposition occur simultaneously. Briefly, in the electrodeposition technique, a precursor solution is prepared and used as the electrolyte. Then, by applying an electric field in the electrolyte in an electrochemical cell, cations move toward the cathode and deposit through a reduction reaction. The electrolyte is usually a modified Watts bath in the electrodeposition synthesis of nickel sulfides and phosphides. The conventional Watts bath, which is commonly used for nickel electroplating, consists of NiSO_4_·7H_2_O, NiCl_2_·6H_2_O, and H_3_BO_3_. For modification, sulfur and phosphorus precursors are added to the bath. Various precursors are used as the sulfur source such as thiourea (SC(NH_2_)_2_),^15–21^ sodium thiocyanate (NaSCN)^22^ and sodium thiosulfate (Na_2_S_2_O_3_·5H_2_O).^23^ However, in the case of the phosphorus precursor, NaH_2_PO_2_·H_2_O is generally always used.^24–31^ In addition to sulfur and phosphorus precursors, it is common to use salts such as NaCl,^16–20,24,25^ KCl^21^ and NaOAc (sodium acetate)^26,27^ to increase the conductivity of the electrolyte. However, the Watts bath and modifiers only ease the synthesis procedure, and in general, it is possible to synthesize nickel sulfides and phosphides *via* the electrodeposition method using a solution of only nickel and sulfur or phosphorus precursors. For example, Cao *et al.*^31^ synthesized Ni–P on nickel foam using an aqueous solution containing only NiCl_2_·6H_2_O and NaH_2_PO_2_·H_2_O as the nickel and phosphorus precursor, respectively.

The parameters affecting the electrodeposition method include the type of electrolysis (galvanostatic, potentiostatic, cyclic voltammetry, *etc.*), current density (in galvanostatic mode), potential (in potentiostatic mode), number of cycles (in cyclic voltammetry mode), concentration of the precursors, electrodeposition time, temperature, pH and additives (conductivity adjusters, capping agents, *etc.*). The effects of these parameters are discussed below.

**Fig. 2:**
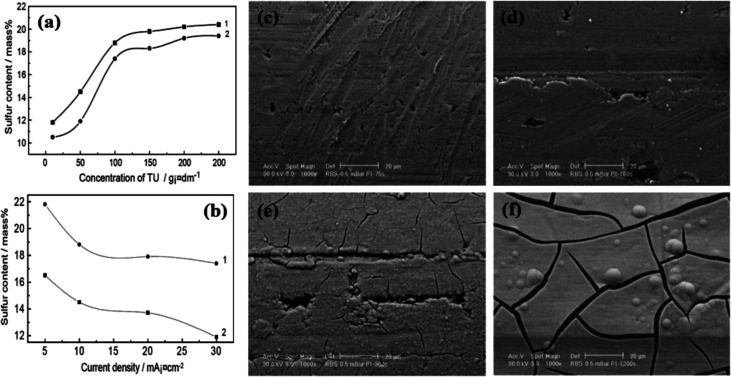
Effect of electrodeposition parameters on the sulfur content of the coating: (a) effect of the thiourea concentration at current densities of (1) 10 mA cm^−2^ and (2) 30 mA cm^−2^ and (b) effect of the electrodeposition current density at thiourea concentrations of (1) 100 g L^−1^ and (2) 50 g L^−1^ (reproduced from ref. 18 with permission from Elsevier, Copyright 2003). SEM images of the Ni–P electrode at different electrodeposition times of: (c) 75 s, (d) 150 s, (e) 300 s and (f) 1200 s (reproduced from ref. 17 with permission from Elsevier, Copyright 2001).

Han *et al.*^18^ investigated the effect of several parameters, including the concentration of the sulfur precursor, current density, pH of the electrolyte, and temperature on the sulfur content of the coating. Fig. 2a shows the effect of thiourea concentration at two different current densities. At concentrations below 100 g L^−1^, the sulfur content of the coating increased sharply as the thiourea concentration increased. However, at concentrations above 100 g L^−1^, the thiourea concentration had little effect on the sulfur content of the coating. A similar result also was obtained by Paseka.^16^

Fig. 2b shows the effect of electrodeposition current density in the range of 5 to 30 mA cm^−2^ on the sulfur content of the deposited nickel sulfide. The sulfur content decreased with an increase in current density. This is because of the different polarizability of the Ni^2+^ ion and its complex with thiourea (Ni[CS(NH_2_)_2_]^2+^) at high current densities, leading to different deposition rates, and finally, a decrease in sulfur content. This result was also reported by Zhang *et al.*^32^ In the following, Han and coworkers investigated the effect of pH and temperature. They found that an increase in pH decreased the sulfur content due to the co-deposition of hydride ions at pH above 4. Also, an increase in temperature favored the dissolution of the deposited nickel and slightly increased the sulfur content deposited. Among these four factors, thiourea concentration and current density had the greatest effect on the sulfur content of the nickel sulfide coating, while pH and temperature had little effect on it. Anyway, the electrodeposition of nickel sulfides and phosphides is usually conducted in acidic media because it is assumed that sulfur and phosphorus tend to be electrodeposited more at low pH.

Although the temperature has little effect on the sulfur content of Ni–S electrocatalysts, the phosphorus content of Ni–P electrocatalysts dramatically depends on temperature. However, there are contradictory reports about how temperature affects the phosphorus content. In this regard, Burchardt^25^ examined the effect of temperature on the phosphorus content of Ni–P electrocatalysts. He reported that by increasing the temperature, the phosphorus content increased, whereas Wasalathanthri *et al.*^30^ reported that a decrease in temperature increased the phosphorus content. Thus, to date, it is only known that temperature affects the phosphorus content of Ni–P electrocatalysts, but it is not clear how it is influenced by temperature, which needs to be further investigated in future works.

Electrodeposition time is another parameter affecting the electrodeposition method. The electrodeposition time can affect the property of electrocatalysts by increasing their thickness and changing the morphology of the coating. In this regard, Paseka^17^ studied the effect of electrodeposition time on the morphology of Ni–P and Ni–S electrocatalysts. Fig. 2c–f shows the SEM images of Ni–P electrodes synthesized at different electrodeposition times. As can be seen, the amount of surface cracks increased with electrodeposition time. The calculation of the roughness factor for Ni–P and Ni–S electrodes at different electrodeposition times also confirmed this effect, where the increase in electrodeposition time increased the roughness factor of the electrodes.

Although electrodeposition mainly gives rise to amorphous structures, crystalline nickel sulfides have also been synthesized using this method. Current density is one of the critical factors determining the crystalline phase of nickel sulfide synthesized by the electrodeposition method. Zhang *et al.*^32^ synthesized amorphous nickel sulfide, Ni_3_S_2_ and NiS at 45 °C by changing the electrodeposition current density in the range of 6 to 12 mA cm^−2^. At current densities of 12, 10, 8 and 6 mA cm^−2^, amorphous, a mixture of amorphous and Ni_3_S_2_, almost pure Ni_3_S_2_ and a mixture of Ni_3_S_2_ and NiS phases were obtained, respectively. In fact, sulfur-rich phases were obtained at lower current densities. This is because as mentioned, more sulfur is deposited as the electrodeposition current density decreases. In another study, Murthy *et al.*^33^ synthesized a pure NiS crystalline phase *via* an electrodeposition method. They used cyclic voltammetry in the range of −1 to 0.4 V for deposition and reported that the activity of the resulting electrocatalyst in the HER depended on the number of cycles. Accordingly, in their work, the best sample was obtained at 25 cycles. It is worth mentioning that no report was found about the direct synthesis of crystalline phases of nickel phosphide by an electrodeposition method, which should be considered a subject to be studied in future works.

One of the advantages of electrodeposition is the facile synthesis of various alloys and composites. Typically, only a suitable precursor of the desired material is required together with the main precursors. In this regard, the synthesis of Co–Ni–S with CoCl_2_·6H_2_O^18,34,35^ or CoSO_4_·7H_2_O,^36^ Mn–Ni–S with MnSO_4_·2H_2_O,^23^ B–Ni–S with Na_2_B_4_O_7_·10H_2_O^37^ and Ni–P–Ag with AgNO_3_ (ref. 38) are some examples.

### 4.2. Solvothermal route

The solvothermal route is a popular and facile method for the synthesis of various materials, including nickel sulfides and phosphides. Concisely, in the standard solvothermal method, a solution of precursors is heated in an autoclave at a certain temperature for a specific time. If the solvent is water, this method also is called hydrothermal synthesis. The synthesis of nickel sulfides and phosphides *via* the solvothermal method has been conducted with water (hydrothermal method)^39–47^ and other solvents such as ethanol,^48^ oleylamine,^49^ ethanolamine,^50^ ethylene glycol,^51^ triethylene glycol,^52^ ethylenediamine^53^ and mixed solvents.^54–59^ The most used nickel precursor is nickel chloride,^39,41,42,47,54,56,57^ while other precursors such as nickel nitrate,^39,59,60^ nickel acetate^44,45,49,55,61–63^ and nickel foam^39,48,50–53^ have also been used. Similar to the electrodeposition method, for the synthesis of nickel sulfides, different sulfur precursors such as sulfur powder,^40,49,51,61^ thiourea^39,40,62^ and thioacetamide^35^ have been used, but only NaH_2_PO_2_·H_2_O^41,42,56,57,59^ and red phosphorus^43–48,50,52–55,58,60^ have been commonly used as the phosphorus precursor in the synthesis of nickel phosphides.

There are several parameters affecting the solvothermal method, which are mainly synthesis time, temperature, pH, type of precursors, type of solvent, concentration of precursors, and additives (capping agents, reducing agents, shape controller, *etc.*). The effects of these factors, specifically on the synthesis of nickel sulfides and phosphides, are discussed below.

**Fig. 3:**
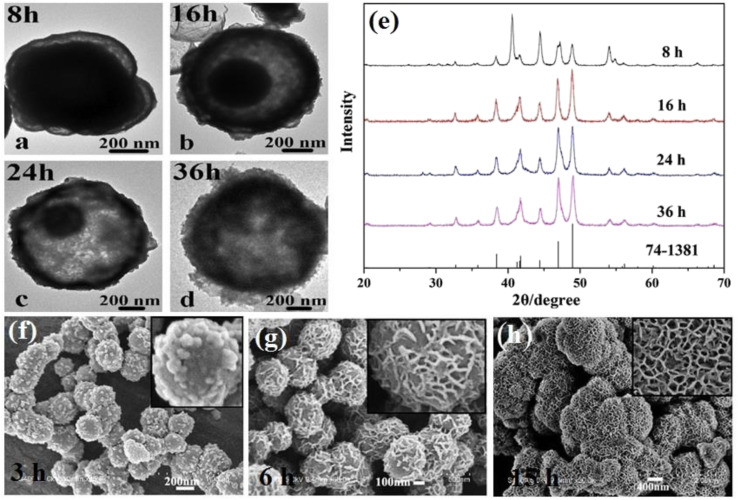
Effect of synthesis time on: (a)–(d) structure, (e) crystalline phase (reproduced from ref. 60 with permission from Elsevier, Copyright 2015), and (f)–(h) morphology of nickel phosphides.^41^

The synthesis time is one of the important parameters in the solvothermal method. The general effect of the synthesis time is through Ostwald ripening. Ostwald ripening is one of the particle growth mechanisms in the wet synthesis methods, including solvothermal route. Based on this mechanism, small particles have high solubility and the potential to dissolve in a solvent as they are synthesized due to their high surface energy. After dissolution, these particles heterogeneously grow on the larger particles with higher stability. The longer the synthesis time, the more these smaller particles dissolve and redeposit, leading to the formation of larger particles. Thus, one of the effects of prolonging the synthesis time in the solvothermal method is the formation of larger particles. It is worth mentioning that the Ostwald ripening mechanism, in addition to increasing the average particle size, also narrows the particle size distribution. Beside these general effects, the reaction time can also change the crystalline phase of the product. In the solvothermal synthesis of nickel phosphides, it seems that a longer reaction time favors the synthesis of Ni_12_P_5_ compared to Ni_2_P. Liu *et al.*^60^ reported that using a surfactant-aided solvothermal method at a synthesis time of 8, 16–24, and 36 h, spherical Ni_2_P, core–shell Ni_2_P/Ni_12_P_5_, and spherical Ni_12_P_5_ were achieved, respectively (Fig. 3a–e). This hypothesis was also seen in the work by Liu *et al.*,^64^ where under identical conditions and different reaction times of 24 and 48 h, almost pure Ni_2_P and Ni_12_P_5_ were achieved, respectively. The synthesis time can also influence the morphology of the product. In this regard, Ni *et al.*^41^ investigated the effect of the synthesis time on the morphology of the Ni_12_P_5_ product. According to Fig. 3f–h, by prolonging the synthesis time from 3 to 15 h, almost smooth spheres were converted to an immature porous structure, and then a compact porous superstructure.

**Fig. 4:**
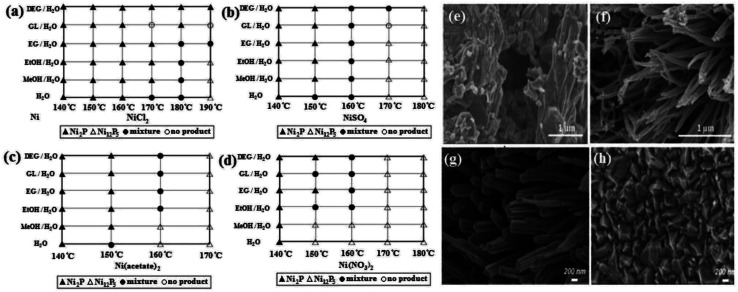
Synthesis of nickel phosphides *via* the solvothermal method with different nickel precursors: (a) NiCl_2_·6H_2_O, (b) NiSO_4_·6H_2_O, (c) Ni(acetate)_2_·4H_2_O and (d) Ni(NO3)_2_·6H_2_O (mixed solvents are a mixture with equal volume ratio and MeOH, EtOH, EG, GL, and DEG stand for methanol, ethanol, ethylene glycol, glycerol and diethylene glycol, respectively).^43^ SEM images of NiCo_2_S_4_: (e) without using nickel foam and (f) with use of nickel foam (reproduced from ref. 66 with permission from Wiley, Copyright 2016). SEM images of (g) V-doped Ni_3_S_2_ using Na_3_VO_4_·12H_2_O and (h) Ni_3_S_2_ (reproduced from ref. 68 with permission from the American Chemical Society, Copyright 2017).

Temperature is another effective parameter in the solvothermal method. An increase in temperature directs the synthesis to the nucleation and formation of small particles. For example, Liu *et al.*^47^ showed that increasing the reaction temperature from 120 °C to 200 °C decreased the average particle size of Ni_2_P from 200 to 20 nm. In addition to the decrease in particle size, synthesis at higher temperatures has more potential to provide the needed crystallization energy, causing the growth of more grains. This can be seen in the sharper peaks of the XRD patterns, as was reported in work by Wang *et al.*,^65^ in which the crystallite size of bimetallic sulfide CoNi_2_S_4_ was calculated using the Scherrer equation, which increased from 9.3 to 14.3 nm with an increase of temperature from 160 °C to 240 °C. Generally, the solvothermal synthesis of nickel sulfides and phosphides is mainly conducted between 120 °C to 180 °C. Temperature can also change the crystalline phase of the product. In the solvothermal synthesis of nickel phosphides, it seems that higher temperatures are conducive to the synthesis of Ni_12_P_5_ compared to Ni_2_P. In a comprehensive study, Deng *et al.*^43^ investigated the synthesis of nickel phosphides *via* the solvothermal method under different conditions. They showed that with the use of different nickel precursors and different mixed solvents, an increase in temperature favored the synthesis of Ni_12_P_5_ relative to Ni_2_P (Fig. 4a–d). This was also observed in the work by Menezes *et al.*,^44^ where at constant conditions and different synthesis temperatures of 200 °C and 140 °C, Ni_12_P_5_ and Ni_2_P were formed, respectively.

The pH of the solution is also one of the parameters affecting the solvothermal method. This parameter is crucial, especially in synthesizing nickel phosphides with NaH_2_PO_2_·H_2_O. This is because when NaH_2_PO_2_·H_2_O is dissolved in the solution, it dissociates into H_2_PO_2_^1−^ and Na^+^. H_2_PO_2_^1−^ needs OH^−^ to produce PH_3_, which will react with Ni^2+^ to form nickel phosphides. In this regard, Ni *et al.*^41^ found that at pH below 4, no product was obtained, and thus they used NaHCO_3_ as the pH adjuster. The reactions for the synthesis of Ni_2_P and Ni_12_P_5_ with NaH_2_PO_2_·H_2_O are as follows:

PH_3_ generation reaction:

14 3H_2_PO^−^_2_ + OH^−^ → PH_3_ + 2HPO^2−^_3_ + H_2_O

Nickel phosphide formation reactions:

15 12Ni^2+^ + 5PH_3_ + 9e^−^ → Ni_12_P_5_↓ + 15H ^+^

16 2Ni^2+^ + PH_3_ + e^−^ → Ni_2_P↓ + 3H^+^

The direct deposition of catalysts on substrates without the use of a binder is one of the strategies to improve the ion/electron transfer rate because polymer binders such as Nafion and PVDF block the active sites and reduce the activity of the electrocatalysts. In the solvothermal method, if a suitable substrate such as nickel foam is placed directly in the autoclave, the synthesis and deposition coincide, and there is no need to use polymer binder. Nickel foam is one of the substrates with the potential to be used as both a substrate and nickel precursor.^39,48,50–53^ Moreover, nickel foam provides the opportunity to synthesize one-dimensional structures. Sivanantham *et al.*^66^ synthesized NiCo_2_S_4_ nanowire arrays using nickel foam as the substrate *via* a two-step solvothermal method. They reported that nickel foam was necessary for the growth of the one-dimensional structure because when NiCo_2_S_4_ was synthesized without nickel foam, an aggregated and irregular structure was obtained (Fig. 4e and f).

Similar to electrodeposition, the solvothermal method also has high ability to synthesize various complex compounds. Dhandapani *et al.*^67^ synthesized N-doped carbon-embedded Ni_3_S_2_ (Ni_3_S_2_/NC) nanocubes *via* the solvothermal method. They synthesized Ni_3_S_2_/NC by adding N-doped carbon dots together with Ni_3_S_2_ particles to an autoclave. Yu *et al.*^52^ simply coated Ni_8_P_3_ nanosheets with carbon using glucose as the carbon source. In another work, Qu *et al.*^68^ synthesized vanadium-doped Ni_3_S_2_ nanowire arrays *via* the solvothermal method. They found that using Na_3_VO_4_·12H_2_O salt, in addition to vanadium doped in the structure of Ni_3_S_2_, caused the growth of one-dimensional nanowires. This is because, under the same conditions and without using vanadium precursor, they observed the formation of Ni_3_S_2_ triangle-shaped nanoparticles (Fig. 4g and h). This result was also reported by Shang *et al.*^69^

### 4.3. Thermal decomposition

Thermal decomposition is a method for synthesis based on the decomposition of materials in which the precursors are heated to a specific temperature to break the existing chemical bonds and create new ones. In the thermal decomposition synthesis of nickel sulfides and phosphides, usually, nickel(ii) acetylacetonate,^70–79^ Ni(NO_3_)_2_·6H_2_O,^80^ nickel(ii) acetate (Ni(OAc)_2_·4H_2_O)^81,82^ and nickel foam^83^ as the nickel precursor, 1-dodecanethiol,^84^ cysteamine^85^ and thioacetamide^80,83^ as the sulfur precursor, trioctylphosphine (TOP),^71–73,77–79,82,86^ trioctylphosphine oxide (TOPO)^70^ and both of them^74–76,81^ as the phosphorus precursor, and oleylamine (OAm),^84^ 1-octadecane (ODE), pentanediol,^85^*n*-octyl ether,^70,75^ and triethylene glycol^80^ as the solvent have been used.

The thermal decomposition synthesis of nickel sulfides and phosphides is usually carried out at temperatures of around 280 °C and 320 °C, respectively. This is because at these temperatures, the sulfur–carbon (S–C) and phosphorus–carbon (P–C) bonds in the organic precursors are broken.^73,80,84,85^ Thus, the temperature is usually a known and constant value in the thermal decomposition synthesis of nickel sulfides and phosphides. Accordingly, other factors such as the precursor ratio, type of solvent, synthesis time, injection rate, and additives are adjusted to synthesize materials with desired properties.

**Fig. 5:**
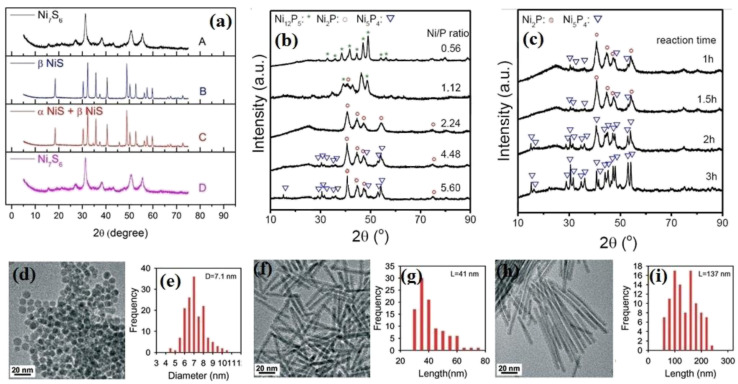
(a) XRD pattern of nickel sulfides at different conditions: (A) Ni : S = 0.18 in OAm, (B) Ni : S = 0.08 in OAm, (C) Ni; S = 0.18 in ODE, and (D) Ni : S = 0.18 in OAm.^84^ (b) XRD pattern of nickel phosphides at different precursor ratios and synthesis time of 2 h. (c) XRD pattern of nickel phosphides at different synthesis times and precursor ratio of (P/Ni) = 4.48 (reproduced from ref. 71 with permission from Wiley, Copyright 2018). SEM images and corresponding particle diameter (for spheres) or length (for nanorods) distributions of nickel phosphides at different injection rates: (d) and (e) all in one pot, (f) and (g) dropwise, and (h) and (i) with lower rate injection than that of (f) and (g).^75^

As mentioned, the precursor ratio and type of solvent are two parameters affecting the thermal decomposition method. Pan *et al.*^84^ investigated the effect of these factors on the structure of the synthesized nickel sulfides. According to their result, at the constant condition of 280 °C and 5 h synthesis time, the crystalline phase of the synthesized nickel sulfides depended on the nickel to sulfur precursor ratio (Ni : S) and the type of solvent. Consequently, by using OAm and decreasing the Ni : S ratio from 0.36 to 0.08, the crystalline phase changed from Ni_7_S_6_ to β-NiS. By lowering this ratio, more sulfur atoms diffused from the sulfur precursor to the nickel precursors, leading to the more sulfur-rich phase of NiS. Regarding the solvent effect, at a constant Ni : S ratio of 0.18 using OAm and octadecane (ODE), Ni_7_S_6_ and a mixture of α-NiS and β-NiS were obtained, respectively. This result can be ascribed to the different capping abilities of these solvents. ODE has weaker capping ability than OAm, leading to the more effortless transfer of sulfur atoms and the formation of NiS (Fig. 5a). The effect of the precursor ratio also has been investigated in the synthesis of nickel phosphides. Pan *et al.*^73^ synthesized different crystalline phases of nickel phosphide at constant conditions and just by changing the molar ratio of TOP, as the phosphorus precursor, to Ni(acac)_2_, as the nickel precursor. Although they could synthesize pure Ni_12_P_5_ and Ni_2_P by changing the precursor ratio, pure Ni_5_P_4_ was not obtained in their experiments even by increasing the precursor molar ratio from 2.18 to 8.75. In another work, Li *et al.*^71^ completed the previous work and investigated the effect of both precursor ratio and synthesis time on the crystalline phase of the obtained nickel phosphide. They reported that when the molar ratio of TOP to Ni(acac)_2_ was 0.56, the product was pure Ni_12_P_5_. By increasing this ratio from 0.56 to 1.12, the pure Ni_12_P_5_ was converted to a mixture of Ni_12_P_5_ and Ni_2_P. A further increase in the ratio to 2.24 resulted in pure Ni_2_P. However, although at the ratio of 4.48, some weak peaks of Ni_5_P_4_ appeared, pure Ni_5_P_4_ was not achieved even by increasing the ratio to 5.6 (Fig. 5b). This showed that the desired crystalline phase was not achieved only by increasing the precursor ratio. Thus, they also investigated the effect of synthesis time, where they found that at the constant precursor ratio of 4.48 and increasing the synthesis time from 1 to 2 h, a mixture of Ni_2_P and Ni_5_P_4_ turned to pure Ni_5_P_4_ (Fig. 5c). Thus, it can be proposed that a higher P/Ni precursor ratio and longer synthesis time are favorable for synthesizing phosphorus-rich nickel phosphides.

The injection rate of the precursors is another parameter affecting the thermal decomposition method. Li *et al.*^74^ reported that when a mixture of Ni(acac)_2_ and TOP was injected into a solution of TOPO in one pot at 330 °C, small particles with a diameter of around 10 nm were obtained, whereas when the mixture was injected dropwise in 1 h into the TOPO solution, nanorods with a length of up to 30 nm and an average diameter of 3 nm were achieved. In another work, Seo *et al.*^75^ synthesized nanospheres and nanorods of Ni_2_P by changing the injection rate. Ni_2_P nanospheres were obtained by adding all the TOP into a solution containing Ni(acac)_2_, while Ni_2_P nanorods were synthesized by continuous injection of a mixture of Ni(acac)_2_ and TOP into the TOPO solution. They also changed the nanorod length by adjusting the injection rate, and thus a lower injection rate favors the synthesis of longer nanorods (Fig. 5d–i). In a similar work, Chung *et al.*^76^ obtained short and long Ni_2_P nanowires by using this effect. Thus, it can be concluded that lower injection rates facilitate the formation of one-dimensional structures.

Reducing agents can ease the synthesis of nickel sulfides and the formation of more sulfur-rich phases. Zheng *et al.*^80^ synthesized NiS, NiS_2_, and Ni_3_S_2_ phases *via* a reducing agent-assisted thermal decomposition method. They synthesized NiS and NiS_2_ with a constant amount of precursors and exploited hydrazine hydrate (N_2_H_4_·H_2_O) as the reducing agent in the synthesis of NiS_2_ to increase the reducing ability of the solvent (triethylene glycol). Triethylene glycol has low reducing ability because the electron in the reduction of thioacetamide into S_2_^2−^ is produced through the slow oxidation of its hydroxyl groups into aldehydes. By using N_2_H_4_·H_2_O as the reducing agent, an [S–NHHN–S]^2−^ complex is formed, which at high temperatures easily turns to S_2_^2−^ and causes the synthesis of a more sulfur-rich NiS_2_ phase.

### 4.4. Vapor–solid reaction

The vapor–solid reaction (VSR) is a standard method for synthesizing metal sulfides and phosphides, especially sulfur-rich and phosphorus-rich samples. In this method, sulfurization or phosphorization is typically conducted by heating a suitable sulfur or phosphorus precursor in a tube furnace to increase the vapor pressure of the precursor or to decompose it and create a sulfurizing or phosphorizing vapor. Then, the vapor is transferred to the nickel precursor location by flowing an inert/carrier gas such as nitrogen or argon inside the tube furnace. The vapor reacts with the solid-state nickel precursor (in general, metal precursor) and forms nickel sulfides or phosphides. Thus, this method is called vapor–solid reaction (wrongly, sometimes this method is called chemical vapor deposition (CVD)).

This method always uses sulfur powder as the sulfur precursor. However, according to the synthesis procedure explained above, other sulfur precursors that have sufficient vapor pressure at moderate temperatures or sulfur precursors will be decomposed to a gas mixture containing sulfurizing components have the potential to be used as sulfur precursors. For example, Li *et al.*^87^ used a sulfur-containing resin and synthesized Ni_3_S_2_ nanowires *via* the VSR method. Regarding the phosphorus precursors, similar to the solvothermal method, only NaH_2_PO_2_·H_2_O and elemental red phosphorus were used as the phosphorus precursors. However, again, based on the VSR synthesis procedure, it seems that other phosphorus precursors with an appropriate vapor pressure at not too high temperature or decomposable phosphorus precursors that decompose to a gas mixture contain a phosphorizing component by heating to a specific temperature can also be used as potential phosphorus sources.

The VSR synthesis of nickel sulfides and phosphides can be carried out through one-step or two-step synthesis. In the one-step synthesis, nickel foam is used as the nickel precursor. In the two-step synthesis, initially, a nickel precursor (mostly Ni(OH)_2_) is synthesized mainly *via* the solvothermal method, but various methods such as electrodeposition,^88^ precipitation^89–91^ and electrospinning^92^ can generally be used. Then, the nickel precursor is placed in a tube furnace for sulfurization or phosphorization in the second step. These two routes each have advantages and disadvantages. For example, one-step VSR synthesis is more straightforward and shorter due to having one less step, but it is limited to the use of nickel foam as the nickel precursor and has limited flexibility to alter the morphology of the product. Alternatively, two-step synthesis can enable the synthesis of nickel sulfides and phosphides from different nickel precursors, synthesis of various composites, and tuning the morphology of the nickel precursor and the final product; however, it needs one more step, which makes the whole procedure more complicated and longer.

**Fig. 6:**
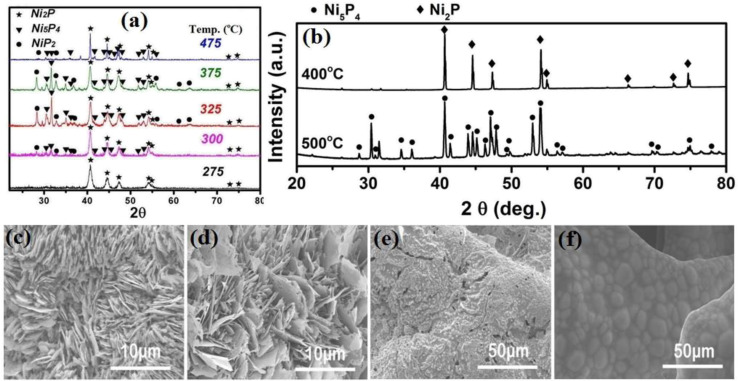
(a) XRD patterns of nickel phosphides at different temperatures (reproduced from ref. 90 with permission from the American Chemical Society, Copyright 2016) and (b) XRD patterns of nickel phosphides at different temperatures (reproduced from ref. 95 with permission from Wiley, Copyright 2015). SEM images of nickel phosphides at different temperatures of: (c) 400 °C, (d) 500 °C, (e) 600 °C and (f) 800 °C (reproduced from ref. 95 with permission from Wiley, Copyright 2015).

The main parameters affecting the VSR method are temperature, synthesis time, position of the precursors, amount of precursors, and type of precursors. The effects of these factors, in particular, on the VSR synthesis of nickel sulfides and phosphides are discussed in the following. Temperature plays a crucial role in the VSR method. As the temperature increases, the vapor pressure of the sulfur or phosphorus precursor increases, which results in a higher degree of sulfurization and phosphorization and the formation of more sulfur- or phosphorus-rich crystalline phases. Also, some materials with multi-step decomposition reactions experience the complete decomposition and generation of sulfurizing and phosphorizing gases at higher temperatures. For example, the decomposition of NaH_2_PO_2_ occurs in three steps, where the first step is triggered at about 310 °C and 0.3 mol PH_3_ is generated per 1 mol of NaH_2_PO_2_. Upon increasing the temperature to 450 °C, complete decomposition occurs, in which 0.4 mol PH_3_ is generated per 1 mol of NaH_2_PO_2_.^93^ Although the increase in temperature facilitates the formation of sulfur- and phosphorus-rich phases, very high temperatures can cause them to decompose again into nickel-rich phases. Thus, there is an optimum value for VSR temperature in the synthesis of sulfur- or phosphorus-rich phases. In this regard, Liu *et al.*^94^ reported that when the VSR synthesis using red phosphorus was conducted at 400 °C, almost no phosphorization occurred, which shows that this temperature was not as sufficient to appropriately sublime red phosphorus. However, nickel phosphides were formed when they increased the synthesis temperature to 500 °C and above it. In another work, Li *et al.*^90^ synthesized various nickel phosphides *via* the VSR method at different temperatures. They reported that pure Ni_2_P was achieved at 275 °C, and by increasing the temperature to 300 °C, a composite with the major content of Ni_2_P and minor contents of Ni_5_P_4_ and NiP_2_ was synthesized. An increase in temperature to 325 °C resulted in a mixture with a higher amount of Ni_5_P_4_ and NiP_2_ and lower content of Ni_2_P. However, a further increase in temperature to 375 °C gave rise to a mixed-phase of Ni_2_P, Ni_5_P_4_ and NiP_2_ with a higher content of Ni_2_P, and finally a higher synthesis temperature of 475 °C further increased the content of Ni_2_P (Fig. 6a).

In a similar work, Wang *et al.*^95^ synthesized nickel phosphides *via* the VSR method and examined the effect of temperature. They observed that by increasing the VSR temperature from 400 °C to 500 °C, the product changed from a mixture of Ni_2_P and Ni_5_P_4_ with higher content of Ni_2_P to a mixture of Ni_2_P and Ni_5_P_4_ with *ca.* 80 wt% of Ni_5_P_4_ (Fig. 6b). However, a further increase in temperature to 600 °C and 800 °C decreased the content of Ni_5_P_4_ to values even lower than that at 400 °C. They also observed that temperature could change the morphology in such a way that at temperatures of 400 °C and 500 °C, nanosheet arrays were formed, while an increase in temperature from 400 °C to 500 °C promoted the growth of nanosheets. However, a further increase in temperature to 600 °C and 800 °C caused the nanosheets to disappear (Fig. 6c–f). The effect of VSR temperature on morphology was also reported by Mishra *et al.*^96^ and Li *et al.*,^87^ where again it was confirmed that an increase in temperature promotes the growth of nanostructures. It should be noted that in the VSR synthesis of nickel phosphides using red phosphorus, after their synthesis, firstly, the tube furnace should be cooled to as low as about 250 °C and maintained at this temperature for a few hours to ensure that the highly reactive and flammable yellow phosphorus is converted to safe red phosphorus.^88^

**Fig. 7:**
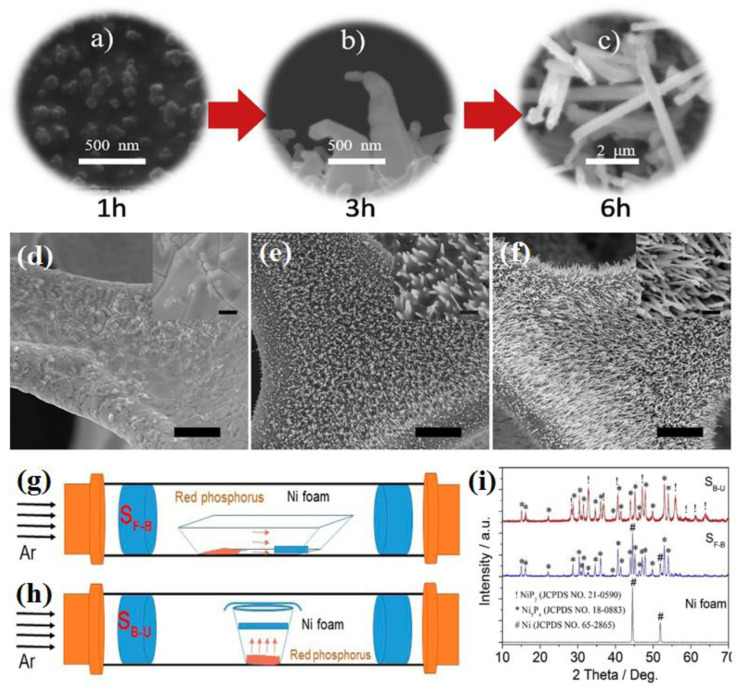
(a)–(c) SEM images of nickel sulfide at different synthesis times (reproduced from ref. 87 with permission from Elsevier, Copyright 2019). (d)–(f) SEM images of nickel foam at different synthesis times: all bars are 20 μm (inset: the corresponding enlargement, all bars are 2 μm).^97^ Schematic of precursor positions in the VSR method: (g) back-front, (h) bottom-up and (i) XRD pattern of nickel phosphides at different positions of the precursors (reproduced from ref. 94 with permission from Elsevier, Copyright 2019).

The synthesis time in the VSR method can affect the crystalline phase and morphology of the products. A longer synthesis time provides the opportunity for complete sulfurization or phosphorization of the nickel precursor and the growth of the formed structures. Specifically, increasing the synthesis time increases the probability of the formation of phases with a higher content of sulfur and phosphorus, and also in the case of one-dimensional structures, it can lengthen them. Li *et al.*^87^ investigated the effect of the synthesis time on the morphology of the product. They reported that when the synthesis was conducted for 1 h, nano-seeds of Ni_3_S_2_ were formed on nickel foam. An increase in the synthesis time to 3 h led to the appearance of Ni_3_S_2_ nanowires, and finally performing synthesis for 6 h caused them to grow further (Fig. 7a–c). This result was also reported by Xiao *et al.*,^97^ who saw that by extending the synthesis time, the nickel phosphide nanowires gradually grew along the vertical direction of the Ni foam (see Fig. 7d–f). In another work, Mishra *et al.*^96^ reported that the synthesis time affects the progress of the phosphorization process in such a way that in their work, according to the amount of red phosphorus, 1 h was not sufficient for the complete phosphorization of nickel foam, and peaks related to nickel were observed in the XRD pattern. By increasing both the content of red phosphorus and synthesis time to 6 h, the nickel foam was turned entirely to nickel phosphide.

In the VSR method, the position of the precursors in the tube furnace is also a factor affecting the synthesis. In most cases, the sulfur or phosphorus precursors are put upstream, while the nickel precursors are placed at the end or middle of the tube furnace (back-front position) (Fig. 7g). However, Li *et al.*^87^ synthesized Ni_3_S_2_ nanowires *via* VSR by placing nickel foam above a sulfur-bearing resin (bottom-up position) (Fig. 7h) or some works^89,98–100^ synthesized different nickel phosphides by preparing a mixture of nickel precursor and NaH_2_PO_2_ and putting the whole mixture in one location. In this regard, Liu *et al.*^94^ investigated the effect of the precursor position in the VSR method. They synthesized mixed-phase nickel phosphides in both back-front and bottom-up positions. The results showed that under the same conditions, the bottom-up position led to the complete phosphorization of nickel foam, whereas in the back-front position, phosphorization was not entirely performed, and there were peaks related to nickel in the XRD pattern of the sample (Fig. 7i). It is worth mentioning that the sample prepared in the bottom-up position had higher HER activity compared to the sample synthesized in the back-front position. According to the above discussion, the advantages and disadvantages of these synthesis methods are summarized in Table 1.

**Table 1:** Comparison of different synthesis methods

Synthesis method	Advantages	Disadvantages
Electrodeposition	Low temperature	Low crystallinity and amorphous products
	Flexibility in precursor selection	Limitations in the synthesis of various morphologies
	Ability to synthesize various composites	Difficulties in conducting powder characterization tests
	Short synthesis time	Limited to conductive substrates
	Binder-free	
	Facile control of loading	
Solvothermal	Flexibility in precursor selection	Relatively long synthesis time
	Facile synthesis procedure	Requires binder for the preparation of electrocatalyst (except cases that use particular substrates inside autoclave)
	Ability to synthesize various morphologies	
	Ability to synthesize various composites	
	Ability to synthesize various crystalline phases	
Thermal decomposition	High crystallinity of products	High temperature
	Ability to synthesize various crystalline phases	Limited to organic sulfur and phosphorus precursors
	Ability to synthesize various composites	Requiring inert atmosphere
	Ability to synthesize various morphologies	Requiring binder for the preparation of electrocatalysts
		Requiring a relatively complicated setup
		Poor HSE
VSR	High crystallinity of products	High temperature
	Ability to synthesize various composites (in two-step synthesis)	Requiring inert/carrier gas
	Binder-free (except for some two-step methods)	Limitations in the synthesis of various morphologies (in one-step synthesis)
	Relatively short synthesis time (in one-step synthesis)	Requiring a relatively complicated setup
		Poor HSE

## 5. Performance in the HER

In this section, we investigate the performance of nickel sulfide and phosphide electrocatalysts in the HER. The main aim is to introduce the most active materials. In addition, the activation step in nickel sulfide electrocatalysts and the performance of nickel phosphide-based composites are also considered in detail. It should be noted that what is explained for the activation step of nickel sulfides and the effects of various materials on the properties of pristine nickel phosphides are also valid for nickel sulfide, and phosphide electrocatalysts and are not presented separately to refrain from repetition. Here, we provide a good view for finding materials with the best performance and illustrate how different materials can affect the performance of pure nickel sulfide and phosphide electrocatalysts.

### 5.1. Nickel sulfide electrocatalysts

**Fig. 8:**
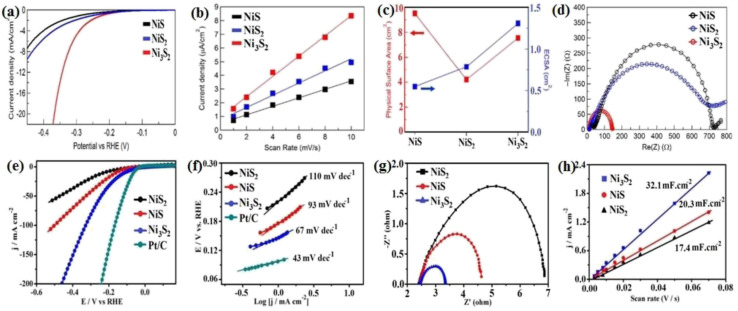
Ni_3_S_2_, NiS and NiS_2_ electrocatalysts: (a) LSV curves in 1.0 M KOH, (b) electrochemical double-layer capacitance, (c) comparison of SSA (red) and ECSA (blue),^49^ (d) EIS, (e) LSV curves in 1.0 M KOH, (f) corresponding Tafel plots, (g) EIS plots and (h) electrochemical double-layer capacitance.^80^

The performance of nickel sulfide electrocatalysts in the HER dramatically depends on their crystalline phase. In fact, their different properties compared to pure nickel can be attributed to their crystalline structure, not just the presence of sulfur.^16^ According to the literature, Ni_3_S_2_ appears to be the most active crystalline phase of nickel sulfides in alkaline media. In this regard, Jiang *et al.*^49^ investigated the HER performance of Ni_3_S_2_, NiS_2_, and NiS on a carbon substrate in 1.0 M KOH. According to Fig. 8a, although all three electrocatalysts were active in the HER, their performance was considerably different. The Ni_3_S_2_ electrocatalyst with the lowest onset potential was the most active sample, which needed an overpotential of −335 mV to deliver a current density of 10 mA cm^−2^. NiS_2_ and NiS required overpotentials of −454 and −474 mV at the same current density, respectively. The better performance of Ni_3_S_2_ compared to NiS and NiS_2_ can be related to the more optimum electrochemical hydrogen adsorption, as was confirmed by the corresponding Tafel plot of the electrocatalysts, where Ni_3_S_2_, NiS_2_ and NiS had Tafel slopes of 97, 128 and 124 mV dec^−1^, respectively (Fig. 8b). The higher conductivity of Ni_3_S_2_ was the second reason for its better performance, where according to the EIS plots, Ni_3_S_2_ with lowest semi-circle diameter had the highest conductivity (Fig. 8d). In addition, Ni_3_S_2_ possessed the largest ECSA among the electrocatalysts. Fig. 8c shows a comparison of the physical and electrochemical surface area of these three electrocatalysts. Although the NiS electrocatalyst had the largest physical surface area, its ECSA was the lowest. Thus, it is not merely important how large the physical surface area of an electrocatalyst is, it is also important how much of the surface is active for the reaction. The percentage of a surface that is active for the reaction can be calculated by dividing the ECSA by the physical surface area, which for the above-mentioned case showed that Ni_3_S_2_ was intrinsically more active than NiS_2_ and NiS in the HER in alkaline media. In a similar study, Zheng *et al.*^80^ considered the HER performance of Ni_3_S_2_, NiS_2_, and NiS on carbon paper in 1.0 M KOH. Based on Fig. 8e, Ni_3_S_2_ was the most active electrocatalyst, which needed *ca.* 50 and 110 mV lower overpotentials than NiS and NiS_2_ for delivering a current density of 10 mA cm^−2^, respectively. This superiority was further confirmed by the Tafel slope of these electrocatalysts (Fig. 8f). To investigate the reasons behind this performance, the ECSA and charge transfer resistance of these electrocatalysts were analyzed. The results showed that Ni_3_S_2_ had the largest ECSA and the lowest charge transfer resistance (Fig. 8g and h), respectively. The better performance of Ni_3_S_2_ in alkaline media also was confirmed by Zhang *et al.*,^32^ where again the better performance of Ni_3_S_2_ was attributed to its optimum electrochemical hydrogen adsorption (lower Tafel slope), higher conductivity and larger ECSA.

Although it seems that Ni_3_S_2_ is the most active crystalline phase of nickel sulfides in alkaline media, comparing the different phases of nickel sulfides in acidic media has been rarely investigated like in the work by Chung *et al.*^85^ Therefore, it is hard to conclude the activity trend of nickel sulfides in acidic media, and further studies have to be conducted in this field in the future.

Besides the crystalline phases, amorphous nickel sulfide electrocatalysts have been applied in the HER even earlier than the crystalline phases since the 1980s. In one of the primary works, in 1983, Vandenborre *et al.*^15^ investigated the performance of amorphous nickel sulfide electrocatalysts in the HER. They found that the thickness of the nickel sulfide layer affected the electrocatalytic performance, where as its thickness increased, the overpotentials decreased. In 2001, Paseka^17^ confirmed this finding. He reported that as the thickness increased, the electrocatalyst activity increased to a limiting thickness, at which the layer decomposed and became mechanically unstable. In another work, Paseka^16^ reported that his synthesized amorphous nickel sulfide was more active than Ni_3_S_2_. He prepared some nickel sulfide electrocatalysts with different sulfur contents on nickel foam *via* an electrodeposition method. Then, the electrocatalysts were heated at 150 °C and their performance in 1.0 M KOH was evaluated before and after heat treatment. The results showed that after heat treatment, the hydrogen adsorption ability of the samples decreased and the needed overpotentials at different current densities increased. The characterization of the heated electrocatalysts showed that the structure of most of them was converted to Ni_3_S_2_ without reducing the sulfur content. Thus, the amorphous nickel sulfides were more active than crystalline Ni_3_S_2_. Although this result does not mean that every amorphous nickel sulfide electrocatalyst necessarily has a better performance than crystalline nickel sulfides such as active Ni_3_S_2_ because amorphous electrocatalysts have specific and different performances due to their random nature, it shows the great potential of amorphous nickel sulfide electrocatalysts in the HER. The sulfur content of amorphous nickel sulfide electrocatalysts is one of the main factors affecting their activity. By increasing the sulfur content to the optimum amount of *ca.* 19%, the HER activity increased and the overpotential decreased, and a further increase in sulfur content lowered the activity of the electrocatalyst. One reason for this phenomenon is the morphological changes. By increasing the sulfur content to more than the optimum amount, the surface roughness of the electrode decreased, consequently lowering the number of exposed active sites and activity.^17–20^

**Fig. 9:**
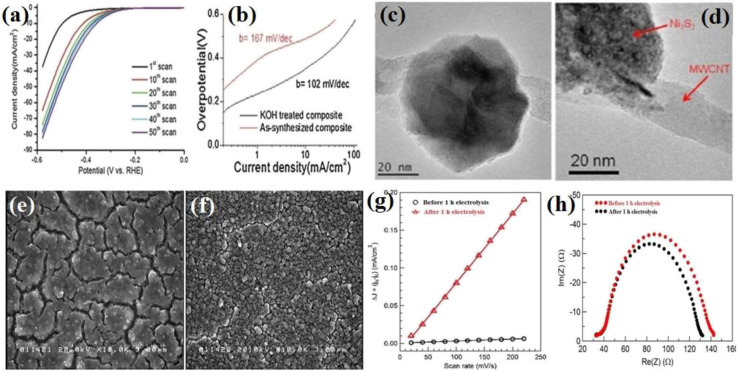
Ni_3_S_2_(55%)/MWCNT-NC: (a) LSV curves at different polarization values in 30 wt% KOH and (b) corresponding Tafel plots before and after 12 h base treatment in 30 wt% KOH. (c) TEM image before base treatment and (d) TEM image after base treatment (reproduced from ref. 101 with permission from Elsevier, Copyright 2014). Ni–S/FTO: (e) SEM image before 1 h electrolysis, (f) SEM image after 1 h electrolysis, (g) electrochemical double-layer capacitance and (h) EIS plots.^21^

Nickel sulfide and phosphide electrocatalysts (in general, all electrocatalysts) usually have an activation step in which their activity is significantly changed. Acid/base treatment or continuous polarization has been performed to study this activation step. In this regard, Lin *et al.*^101^ examined Ni_3_S_2_ loaded on multi-walled carbon nanotube (Ni_3_S_2_(*X*%)/MWCNT) electrocatalysts in which *X* indicates the weight percent of Ni_3_S_2_ in the composites. They immersed the Ni_3_S_2_(55%)/MWCNT electrocatalyst in a 30 wt% KOH solution for 12 h and conducted electrochemical measurements and characterization tests before and after the base treatment. In addition, they investigated the effect of continuous polarization on the activity of the electrocatalysts. Fig. 9a shows the LSV curves of Ni_3_S_2_(55%)/MWCNT at different polarization values. As can be seen, as the number of polarizations increased, the electrocatalyst activity gradually improved to the optimum number, at which the performance of the electrocatalyst became steady. Fig. 9b shows the Tafel plots of Ni_3_S_2_(55%)/MWCNT before and after 12 h of base treatment. It is evident that the KOH-treated electrocatalyst with a lower Tafel slope (102 mV dec^−1^) was more active than the as-synthesized sample (167 mV dec^−1^). They also reported that the base treatment of Ni_3_S_2_(83%)/MWCNT approximately doubled the delivered current density at the overpotential of −580 mV.

To find the cause of this behavior, transmission electron microscopy (TEM) analysis was conducted. According to Fig. 9c and d, the surface of the KOH-treated Ni_3_S_2_(55%)/MWCNT-NC nanoparticle was rougher than the untreated particle. This showed that the base treatment changed the morphology of the electrocatalyst by increasing the surface area and exposed active sites. This finding was further confirmed by calculating the ECSA, where the ECSA of the KOH-treated electrocatalyst was 2-times greater than that of the untreated sample. Also, the EIS result showed that the charge transfer resistance of the KOH-treated electrocatalyst decreased by 7 times. Thus, base treatment and continuous polarization can increase the activity of electrocatalysts by increasing their surface area and exposed active sites and reducing the charge transfer resistance. This observation was also reported in other works.^17,21,39,102^ Jiang *et al.*^21^ showed that the surface cracks of Ni–S/FTO after 1 h of electrolysis in a neutral solution decreased, and the roughness and porosity increased (Fig. 9e and f) in such a way that the ECSA of the electrocatalyst after 1 h of electrolysis got 34 times higher than that of the as-synthesized sample (Fig. 9g). Also, the EIS plots showed that after electrolysis, the conductivity of Ni–S/FTO improved (Fig. 9h). In another work, Ouyang *et al.*^39^ investigated the effect of acid treatment of the nickel foam as the substrate. The results showed that Ni_3_S_2_ on the acid-treated nickel foam was more active than Ni_3_S_2_ on the pristine nickel foam, and again, this improved behavior was attributed to the enhanced surface. A summary of the performance of pure nickel sulfide electrocatalysts is presented in Table 2.

**Table 2:** Summary of the specifications and performances of pure nickel sulfide electrocatalysts in the HER

Catalyst^a^	Substrate	Synthesis method	Electrolyte	Loading^b^ (mg cm^−2^)	*η*_10_ (mV *vs.* RHE)	*b* (mV dec^−1^)	Ref.
Ni–S	Nickel	Electrodeposition	1.0 M KOH	∼4	281	150	35
Ni–S	Nickel foam	Electrodeposition	30% KOH	∼18	190	166	20
NiS	Glassy carbon	Thermal decomposition	0.5 M H_2_SO_4_	0.199	250	51.2	84
NiS	Carbon paper	Thermal decomposition	1.0 M KOH	0.6	160	93	80
NiS	Carbon paper	Solvothermal	0.5 M H_2_SO_4_	0.3	96	52	60
NiS	Glassy carbon	Solvothermal	1.0 M KOH	0.283	474	124	49
NiS	Nickel foam	VSR	1.0 M KOH	43	120	83	103
Ni_3_S_2_	Glassy carbon	Solvothermal	1.0 M KOH	0.283	335	97	49
Ni_3_S_2_	Ni foam	Solvothermal	1.0 M KOH	1.5	123	110	40
Ni_3_S_2_	Ni foam	Solvothermal	1.0 M KOH	19.3	123	92	51
Ni_3_S_2_	Carbon paper	Thermal decomposition	1.0 M KOH	0.6	112	67	80
Ni_3_S_2_	Ni foam	Thermal decomposition	1.0 M KOH	10	199.2	106.1	87
NiS_2_	Glassy carbon	Solvothermal	1.0 M KOH	0.283	335	128	49
NiS_2_	Glassy carbon	Solvothermal	1.0 M KOH	0.7	148	83	104
NiS_2_	Carbon cloth	Solvothermal-VSR	1.0 M KOH	4.1	149	104	105
NiS_2_	Nickel foam	Solvothermal-VSR	1.0 M KOH	1.6	67	72	102
NiS_2_	Carbon cloth	Precipitation-VSR	1.0 M NaOH	1.2	190	85	106
NiS_2_	Carbon paper	Thermal decomposition	1.0 M KOH	0.6	227	110	80

a Ni–S stands for amorphous nickel sulfide.

b For amorphous nickel sulfide, loading indicates the percentage of sulfur in the electrocatalyst.

### 5.2. Nickel phosphide electrocatalysts

**Fig. 10:**
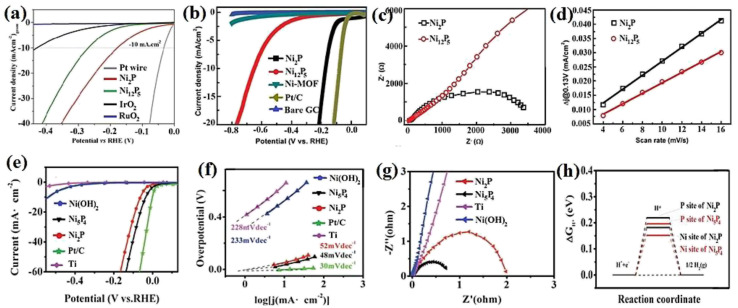
(a) LSV curves of Ni_12_P_5_ and Ni_2_P electrocatalysts on FTO in 1.0 M KOH with loadings of ∼1 mg cm^−2^ (reproduced from ref. 44 with permission from the American Chemical Society, Copyright 2017). MOF-derived Ni_12_P_5_ and Ni_2_P: (b) LSV curves in 0.5 M H_2_SO_4_, (c) EIS plots and (d) electrochemical double-layer capacitance.^98^ Ni_2_P and Ni_5_P_4_: (e) LSV curves in 0.5 M H_2_SO_4_ without *iR* compensation, (f) corresponding Tafel plots, (g) EIS plots and (h) DFT-calculated Gibbs free energy of hydrogen adsorption (reproduced from ref. 109 with permission from Wiley, Copyright 2017).

Nickel phosphides have nine known crystalline phases, including Ni_3_P, Ni_8_P_3_, Ni_5_P_2_, Ni_12_P_5_, Ni_2_P, Ni_5_P_4_, NiP, NiP_2_, and NiP_3_.^107,108^ Similar to nickel sulfides, the performance of crystalline nickel phosphide electrocatalysts in the HER is considerably phase-dependentin such a way that by increasing the phosphorus content of the compound, the HER activity and stability increase. In other words, crystalline phases with a higher phosphorus content show better performances in the HER. In this regard, Menezes *et al.*^44^ investigated the HER activity of Ni_12_P_5_ and Ni_2_P on fluorinated tin oxide (FTO) and nickel foam substrates with different loadings in 1.0 M KOH solution. They found that on both substrates and with different loadings, Ni_2_P was more active than Ni_12_P_5_ (Fig. 10a). This activity trend was also reported by Tian *et al.*,^98^ who investigated MOF-derived Ni_12_P_5_ and Ni_2_P (Fig. 10b). The result of the electrochemical double-layer capacitance and conductivity of the electrocatalysts showed that Ni_2_P had a lower charge transfer resistance and higher electrochemical double-layer capacitance than Ni_12_P_5_, indicating that the better performance of more phosphorus-rich nickel phosphides can be attributed to their higher conductivity and larger electrochemical active surface area (Fig. 10c and d). To investigate more phosphorus-rich phases, Wang *et al.*^109^ studied the HER activity of Ni_2_P and Ni_5_P_4_ on Ti foil in both acidic and alkaline media. They observed that Ni_5_P_4_ with smaller overpotentials and Tafel slope in both media showed better performances than Ni_2_P (Fig. 10e and f). They conducted an EIS test to investigate the reason behind the better performance of Ni_5_P_4_ (Fig. 10g). The results showed that Ni_5_P_4_ had a smaller charge transfer resistance than Ni_2_P. They also theoretically studied the hydrogen adsorption of Ni_2_P and Ni_5_P_4_ through density functional theory (DFT) tools (Fig. 10h). The results showed that the nickel and phosphorus sites in Ni_5_P_4_ had lower and closer to zero Gibbs free energy of hydrogen adsorption than Ni_2_P, which further confirmed the more optimum hydrogen adsorption of more phosphorus-rich nickel phosphides.

**Fig. 11:**
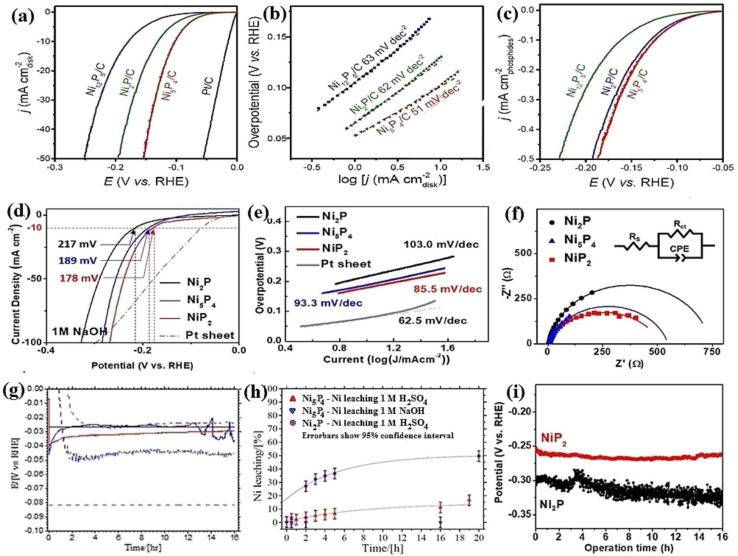
Ni_12_P_5_, Ni_2_P, and Ni_5_P_4_: (a) LSV curves in 0.5 M H_2_SO_4_, (b) corresponding Tafel plots and (c) normalized LSV curves concerning the surface area in 0.5 M H_2_SO_4_ (reproduced from ref. 71 with permission from Wiley, Copyright 2018). Ni_12_P_5_, Ni_2_P and Ni_5_P_4_: (d) LSV curves of in 1.0 M NaOH, (e) corresponding Tafel plots and (f) EIS plots (reproduced from ref. 110 with permission from Elsevier, Copyright 2020). (g) Chronoamperometry test of the Ni_2_P (blue) and Ni_5_P_4_ (red) electrocatalysts: dash lines: 1.0 M NaOH solid lines: 0.5 M H_2_SO_4_, (h) nickel dissolution during chronopotentiometry test and (i)^70^ chronoamperometry test of Ni_2_P and NiP_2_ electrocatalysts (reproduced from ref. 110 with permission from Elsevier, Copyright 2020).

The phase-dependent performance of nickel phosphide electrocatalysts also has been widely investigated by employing more phases of nickel phosphides. In this regard, Li *et al.*^71^ investigated the performance of Ni_12_P_5_, Ni_2_P, and Ni_5_P_4_ electrocatalysts in 0.5 M H_2_SO_4_ (Fig. 11a). They found that the higher the phosphorus content in an electrocatalyst, the better its activity. According to the Tafel plots of the electrocatalysts (Fig. 11b), they attributed the better performance of Ni_5_P_4_ to its better hydrogen binding energy. It is worth mentioning that they also normalized the LSV data relative to the surface area to determine whether the better performance of Ni_5_P_4_ is related to its higher surface area, where again, Ni_5_P_4_ had the best performance, showing that it is intrinsically more active than Ni_2_P and Ni_12_P_5_ (Fig. 11c). Kim *et al.*^110^ employed more phosphorus-rich phases of nickel phosphides and investigated Ni_2_P, Ni_5_P_4_, and NiP_2_ in 1.0 M NaOH. The LSV curves showed that NiP_2_ with lower needed overpotentials than other electrocatalysts was the most active phase (Fig. 11d). As previously proposed, it was seen here again that more phosphorus-rich phases of nickel phosphides had better hydrogen adsorption and higher conductivity according to the Tafel and EIS plots (Fig. 11e and f), respectively.

In addition to activity, the stability and durability of nickel phosphides increase with an increase in phosphorus content in their structure. Laursen^70^ investigated the performance of Ni_2_P and Ni_5_P_4_ pellets in both acidic and alkaline media. In addition to reporting a similar trend for the activity of the above-mentioned crystalline phases, they studied the stability of the electrocatalysts. The results indicated that Ni_5_P_4_ had a stable performance in both media, whereas Ni_2_P showed a relatively unstable performance, especially at a longer electrolysis time (Fig. 11g). To determine the reason for this stability trend, they considered the compositional change of the electrocatalysts after electrolysis, where the results showed that a significant amount of nickel content of Ni_2_P (about 50%) was dissolved in 1.0 M H_2_SO_4_ solution after 20 h of chronopotentiometry test (compared with that of about 16% for Ni_5_P_4_) (Fig. 11h). Thus, the stability of nickel phosphides improves with an increase in phosphorus content in their structure due to their lower compositional changes. In another work, Kim *et al.*^110^ compared the stability of NiP_2_ and Ni_2_P in 1.0 M NaOH. The chronoamperometry test showed that NiP_2_ had better stability than Ni_2_P (Fig. 11i). They also showed that a considerable amount of the Ni_2_P electrocatalyst was dissolved in the electrolyte after 4 h of the chronopotentiometry test.

Considering all the reported data for comparing different crystalline phases of nickel phosphides, it can be concluded that by increasing the phosphorus content of the crystalline phases of nickel phosphides, their activity and stability in the HER increase. The better HER performance of more phosphorus-rich nickel phosphides is mainly attributed to their better electrochemical hydrogen adsorption (lower Tafel slope), higher conductivity (smaller EIS semi-circle), and higher ECSA (higher *C*_dl_). Also, their better stability is related to their more stable compositional and morphological structure.

Although the activity of crystalline nickel phosphide electrocatalysts increases with an increase in the phosphorus content of the crystalline phase with no limitation, this is not the case in amorphous nickel phosphide electrocatalysts. The activity of the amorphous nickel phosphide electrocatalysts increases up to an optimum amount by increasing the phosphorus content, and then decreases. In this regard, Burchardt^24^ investigated the variation in the HER activity of amorphous nickel phosphide as a function of the phosphorus content of the coating. He found that the HER activity of the Ni–P electrocatalysts increased by increasing the phosphorus content up to about 17 at%, and then decreased in such a way that at the overpotential of −1.3 V *vs.* SCE, the delivered current density of the Ni–P electrode with 17.1 at% P was about 20-times greater than the Ni–P electrode with 27 at%. A similar result was also reported in his next study.^25^ However, the phosphorus content-dependence behavior of amorphous nickel phosphides has to be further studied in future works.

Nickel phosphides have high activity toward the HER. Nevertheless, their activity can be enhanced even more by combining specific materials. Various ideas have been implemented in this regard, such as the use of transition metals, heteroatoms, and carbonaceous materials. Next, we present several works on the use of nickel phosphide composites in the HER and reveal how they affect the properties of pristine nickel phosphides.

**Fig. 12:**
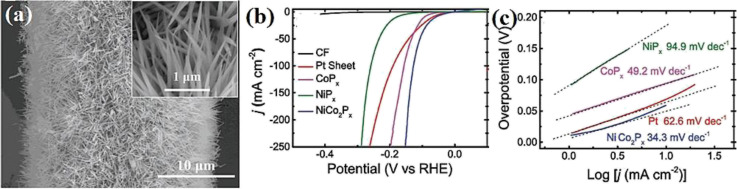
NiCo_2_P_*x*_ nanowire arrays: (a) SEM image, (b) LSV curves in 1.0 M KOH and (c) corresponding Tafel plots (reproduced from ref. 111 with permission from Wiley, Copyright 2017).

Because of their low price and abundant resources, transition metals have received significant attention as a potential substitute for platinum group metals. However, transition metals have no activity comparable to platinum group metals and are usually used in the form of sulfides, phosphides, *etc.* Nevertheless, due to their excellent hydrogen adsorption ability, high conductivity, and active sites toward the HER, they can be employed with active materials such as nickel sulfides and phosphides to improve the performance of the whole system. In this regard, Zhang *et al.*^111^ showed the great potential of transition metals for boosting the HER activity of nickel phosphides, where the incorporation of Co in Ni_2_P, named as self-supported NiCo_2_P_*x*_ nanowire arrays with overpotentials of 11 and 58 mV at current densities of 1 and 10 mA cm^−2^, exhibited an even better performance than Pt sheets in 1.0 M KOH with values of 23 and 70 mV (Fig. 12a and b), respectively. The Tafel plots indicated that NiCo_2_P_*x*_ had the lowest Tafel slopes among Pt sheets, Ni_2_P, and CoP, which means that the better performance of NiCo_2_P_*x*_ can be ascribed to its modified hydrogen adsorption and close to zero Gibbs free energy of hydrogen adsorption (Fig. 12c). Ma *et al.*^112^ further proved the better performance of cobalt-nickel bimetallic phosphide than single metal phosphides of CoP and Ni_2_P in acidic and alkaline media. The EIS plots and electrochemical double-layer capacitances of the electrocatalysts showed that the better performance of cobalt–nickel phosphide could be related to the improved conductivity and enhancement of active sites. A similar result was also reported by Liu *et al.*^113^

**Fig. 13:**
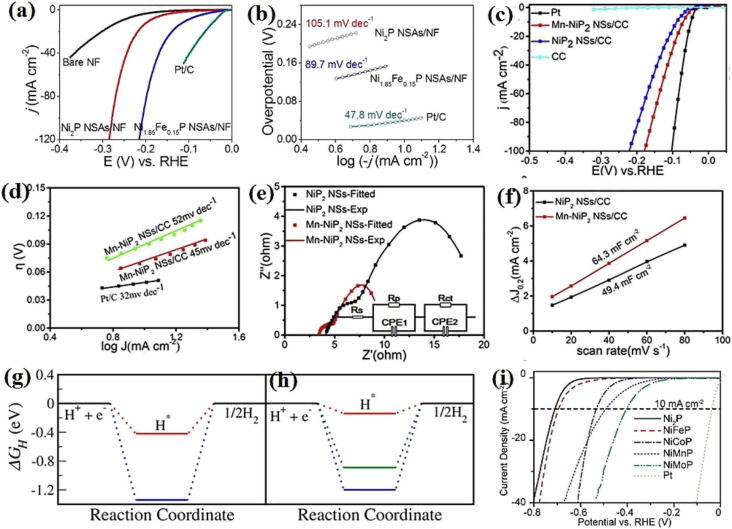
Iron-doped Ni_2_P: (a) LSV curves in 1.0 M KOH and (b) corresponding Tafel plots (reproduced from ref. 114 with permission from the American Chemistry Society, Copyright 2017). Mn-Doped NiP_2_: (c) LSV curves in 0.5 M H_2_SO_4_, (d) corresponding Tafel plots, (e) EIS plots and (f) electrochemical double-layer capacitance (reproduced from ref. 115 with permission from Elsevier, Copyright 2018). Gibbs free energy of hydrogen adsorption of (g) Ni_2_P and (h) Mn-incorporated Ni_2_P (red, blue, and green lines correspond to adsorption at P, Ni, and Mn sites, respectively) (reproduced from ref. 118 with permission from the American Chemistry Society, Copyright 2020). (i) LSV curves of transition metal-doped Ni_2_P (reproduced from ref. 81 with permission from Elsevier, Copyright 2019).

Wang *et al.*^114^ studied the incorporation of iron in nickel phosphide by doping iron in Ni_2_P nanosheet arrays. The results showed that the addition of iron improved both the HER and OER activity of pristine Ni_2_P (Fig. 13a). The Tafel plots indicated that iron doping decreased the Tafel slope and accelerated the HER kinetics by modifying the hydrogen adsorption (Fig. 13b). Huang *et al.* also investigated iron-tuned Ni_2_P and observed similar results. In another work, Wang *et al.*^115^ considered Mn doping of nickel phosphide by synthesizing Mn-doped NiP_2_ nanosheets. The LSV curves showed that Mn doping improved the HER activity of pristine NiP_2_ in a wide pH range from acidic to alkaline media (Fig. 13c). Similar to previous reports in the transition metal-doping of nickel phosphides, Mn doping also accelerated the HER kinetics, lowered the charge transfer resistance, and increased the ECSA (Fig. 13d–f). In similar studies, Zhang *et al.*^116^ and Jiang *et al.*^117^ investigated Mn-doped Ni_2_P and again reported an enhancement in the HER activity of pristine Ni_2_P through just the above-mentioned improvements. The modification of hydrogen adsorption by Mn doping was also theoretically confirmed by DFT calculations. According to Fig. 13g and h, by doping Mn in the structure of Ni_2_P, the Gibbs free energy of hydrogen adsorption of the nickel and phosphorus sites in Ni_2_P approached zero, thus accelerating the kinetics of the HER, and the activity of the electrocatalyst toward the HER increased.^118^ Man *et al.*^81^ investigated the transition metal-doping of nickel phosphides using various transition metals, including Mn, Co, Fe, and Mo, in a comprehensive study. The polarization curves of these electrocatalysts showed that generally, transition metal-doping improved the activity of pristine nickel phosphide (Ni_2_P), where molybdenum had the best and most significant effect on the HER activity of Ni_2_P (Fig. 13i).

**Fig. 14:**
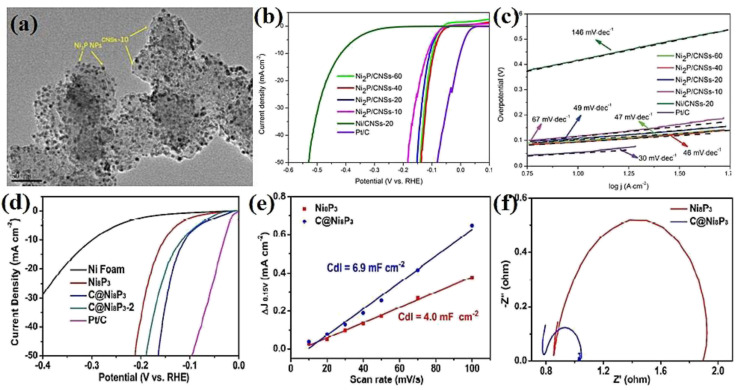
Ni_2_P supported on carbon nanospheres: (a) TEM image, (b) LSV curves in 0.5 M H_2_SO_4_ and (c) corresponding Tafel plots (reproduced from ref. 78 with permission from Elsevier, Copyright 2015). Carbon-coated Ni_8_P_3_: (d) LSV curves in 0.5 M H_2_SO_4_, (e) electrochemical double-layer capacitance and (f) EIS plots (reproduced from ref. 108 with permission from the America Chemistry Society, Copyright 2016).

Due to their high electron transfer ability, high specific surface area, and porous structure, carbonaceous materials are widely used in electrochemical applications, ranging from batteries and supercapacitors to electrocatalysts. However, although carbonaceous materials have high conductivity and surface area, they have no considerable active sites or HER activity. Consequently, they cannot be solely used as electrocatalysts and should be used in combination with active materials. In this regard, Pan *et al.*^78^ loaded Ni_2_P nanoparticles on carbon nanospheres with different loadings (Fig. 14a). The LSV curves and Tafel plots showed that increasing the amount of carbon nanospheres enhanced the activity of Ni_2_P up to an optimum amount (Fig. 14b and c). Although the specific surface area of the electrocatalysts increased by increasing the content of carbon nanospheres according to the calculated BET surface area of the electrocatalysts, the carbon nanospheres had almost no active sites. Thus, there was a trade-off between an increase in activity through the increase of surface area and improvement of conductivity, as was demonstrated by the EIS test, and a decrease in the number of active sites by lowering the content of Ni_2_P as the active material. A similar result was reported by Li *et al.*,^45^ where they used a nanohybrid of Ni_2_P film and carbon nanosheets. Yu *et al.*^108^ coated nanosheets of Ni_8_P_3_ with carbon in two different amounts *via* simple hydrothermal carbonization. The polarization curves showed that carbon coating improved the activity of Ni_8_P_3_, whereas extra carbon coating decreased the activity, which was due to the above-mentioned reason. However, the performance of both carbon-coated electrocatalysts was still better than that of the pristine Ni_8_P_3_ (Fig. 14d). The calculated electrochemical double-layer capacitance of the electrocatalysts showed that the *C*_dl_ increased slightly after the carbon coating, while based on the EIS plots, the main effect of the carbon coating was lowering the charge transfer resistance, as expected (Fig. 14e and f). In another work, Wang *et al.*^119^ electrodeposited amorphous nickel phosphide on a 3D porous carbon nanotube support. The results showed that while the carbon nanotube support had close to zero HER activity and low ECSA, the Ni–P on carbon nanotube support had high activity and ECSA in a wide pH range from acidic to alkaline media, again confirming that carbonaceous materials affect the electrochemical performance of materials through the improvement of electron transfer and surface area.

**Fig. 15:**
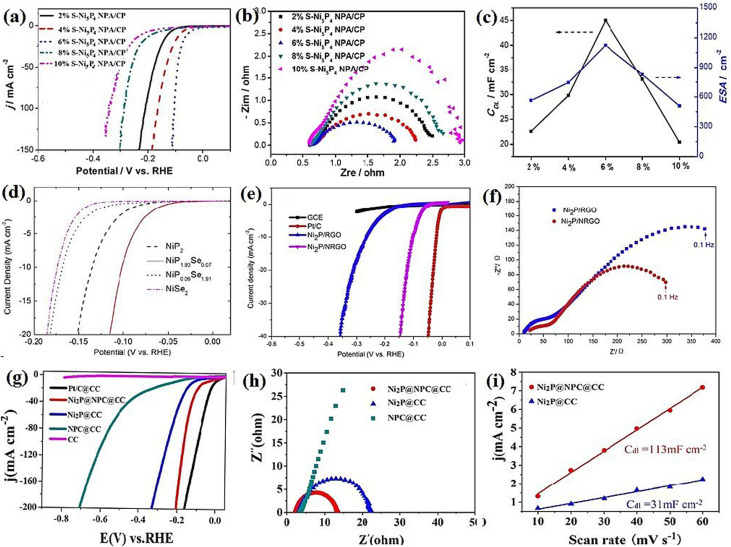
Sulfur-doped Ni_5_P_4_: (a) LSV curves in 0.5 M H_2_SO_4_, (b) EIS plots and (c) ECSA as a function of sulfur content (reproduced from ref. 120 with permission from the America Chemistry Society, Copyright 2018). (d) LSV curves of selenium-doped NiP_2_ (reproduced from ref. 121 with permission from the America Chemistry Society, Copyright 2015). Nickel phosphide nanoparticle–nitrogen-doped graphene hybrid: (e) LSV curves and (f) EIS plots (reproduced from ref. 77 with permission from Elsevier, copyright 2015). Nickel phosphide nanoparticles with nitrogen and phosphorus co-doped porous carbon: (g) LSV curves, (h) EIS plots and (i) electrochemical double-layer capacitance (reproduced from ref. 123 with permission from Elsevier, copyright 2020).

Heteroatoms are another group of materials that can be combined with nickel sulfide and phosphides to improve the HER activity of pristine materials. In organic chemistry, heteroatoms refer to elements other than carbon and hydrogen that can replace carbon in organic molecules. Here, heteroatoms can be considered as nitrogen (N), sulfur (S), phosphorus (P), and selenium (Se). Heteroatoms have shown high ability to boost the activity of transition metals toward the HER, where transition metal phosphides, sulfides, selenides, and nitrides have been shown to be the most promising materials and the main focus of researchers in the field of finding non-noble metal electrocatalysts for the HER. Heteroatoms can be further applied to improve the electrocatalytic properties of materials. In this regard, Chang *et al.*^120^ introduced sulfur-doped Ni_5_P_4_ nanoplate arrays with different sulfur contents as efficient electrocatalysts in acidic HER. The LSV curves showed that the activity increased by increasing the sulfur content from 2% to 6%, and then decreased from 6% to 10%, and thus 6% was the optimum sulfur content (Fig. 15a). The experimental results showed that the best performance of 6% S-doped Ni_5_P_4_ was related to its maximum ECSA, optimum electrochemical hydrogen adsorption (the lowest Tafel slope), and highest conductivity (Fig. 15b and c). DFT calculations further confirmed the superiority of S-doped Ni_5_P_4_ from a theoretical view. It is also worth mentioning that they synthesized pure Ni_5_P_4_ and NiS_2_ for comparison with 6% S-doped Ni_5_P_4_, where it showed a performance even better than pristine Ni_5_P_4_ and NiS_2_. In another work, Zhou *et al.*^121^ investigated selenium-doped NiP_2_ and phosphorus-doped NiSe_2_ and compared their activity with that of pure NiP_2_ and NiSe_2_. The LSV curves showed that the HER activity followed the order of Se-doped NiP_2_ > NiP_2_ > P-doped NiSe_2_ > NiSe_2_ (Fig. 15d), which indicates the positive effect of heteroatom doping, and also the superior activity of the nickel phosphide in comparison with nickel selenide.

Heteroatoms can also be combined with carbonaceous materials, significantly improving the performance of the base materials in various aspects. In this regard, Pan *et al.*^77^ combined Ni_2_P nanoparticles with reduced graphene oxide and nitrogen-doped reduced graphene oxide. The LSV curves indicated that nitrogen doping considerably decreased the onset potential, which could be attributed to the increase in conductivity, as confirmed by the EIS test (Fig. 15e and f). Also, the calculation of the active sites showed that the number of active sites for Ni_2_P on the nitrogen-doped reduced graphene oxide was about 10-times higher than that of Ni_2_P on reduced graphene oxide, which revealed that nitrogen doping has a significant effect on the increase of both conductivity and active sites. This result also was reported by Wang *et al.*^122^ for Ni_2_P embedded in nitrogen-doped carbon nanofibres. In another work, Ma *et al.*^123^ synthesized Ni_2_P nanoparticles on nitrogen and phosphorus co-doped carbon (Ni_2_P@NPC) and evaluated their HER activity in 1.0 M KOH. The polarization curves showed that the use of nitrogen and phosphorus co-doped carbon improved the activity of pristine Ni_2_P and activated the inactive bare carbon cloth (Fig. 15g). The combination of nitrogen and phosphorus co-doped carbon with Ni_2_P improved the conductivity and ECSA, and thus Ni_2_P@NPC had a *C*_dl_ of about 400% higher than that of Ni_2_P nanoparticles (Fig. 15h and i). A summary of the performance of pure nickel phosphide electrocatalysts is presented in Table 3.

**Table 3:** Summary of the specifications and performances of pure nickel phosphide electrocatalysts in the HER

Catalyst^a^	Substrate	Synthesis method	Electrolyte	Loading^b^ (mg cm^−2^)	*η*_10_ (mV *vs.* RHE)	*b* (mV dec^−1^)	Ref.
Ni–P	Nickel foam	Electrodeposition	1.0 M KOH	4.4	63	55	31
Ni–P	Copper foam	Electrodeposition	1.0 M KOH	5	98	55	27
Ni_8_P_3_	Nickel foam	Solvothermal	0.5 M H_2_SO_4_	1.7	152	86	52
Ni_12_P_5_	Glassy carbon	Thermal decomposition	0.5 M H_2_SO_4_	1.99	280	75	73
Ni_12_P_5_	Carbon black	Thermal decomposition	0.5 M H_2_SO_4_	0.12	182	63	71
Ni_2_P	Glassy carbon	Solvothermal	0.5 M H_2_SO_4_	0.429	295	115	45
Ni_2_P	Ti foil	Thermal decomposition	0.5 M H_2_SO_4_	1	78	41.4	82
Ni_2_P	Carbon black	Thermal decomposition	0.5 M H_2_SO_4_	0.12	135	62	71
Ni_2_P	Glassy carbon	Thermal decomposition	0.5 M H_2_SO_4_	0.35	137	49	73
Ni_2_P	Glassy carbon	VSR	0.5 M H_2_SO_4_	0.35	172	62	98
Ni_2_P	Graphite	VSR	1.0 M NaOH	1.7	217	103	110
Ni_2_P	Ti foil	VSR	0.5 M H_2_SO_4_	4.2	56.6	52	109
			1.0 M KOH	4.2	62.2	61	
Ni_5_P_4_	Glassy carbon	Thermal decomposition	0.5 M H_2_SO_4_	1.99	118	42	73
Ni_5_P_4_	Carbon black	Thermal decomposition	0.5 M H_2_SO_4_	0.12	103	51	71
Ni_5_P_4_	Carbon paper	VSR	0.5 M H_2_SO_4_	0.2	95	97.4	120
Ni_5_P_4_	Graphite	VSR	1.0 M NaOH	1.7	189	93.3	110
Ni_5_P_4_	Ti foil	VSR	0.5 M H_2_SO_4_	4.6	35.4	48	109
			1.0 M KOH	4.6	47.1	56	
NiP_2_	Graphite	VSR	1.0 M NaOH	1.7	178	85.2	110
NiP_2_	Carbon cloth	VSR	0.5 M H_2_SO_4_	4.4	84	52	115
			1.0 M KOH	4.4	122	77	

a Ni–P stands for amorphous nickel phosphide.

b For amorphous nickel phosphide, loading indicates the percentage of the sulfur content of the electrocatalyst.

## 6. Challenges and future perspectives

### 6.1. General points for both nickel sulfides and phosphides

#### 6.1.1. Exploiting similarities between hydrotreating and HER

Hydrotreating is a catalytic process in which the contaminants and impurities (sulfur, nitrogen, oxygen, and halides) in the hydrocarbon streams are removed in the presence of hydrogen.^124^ Hydrotreating and the HER have a similar mechanism given that both deal with hydrogen adsorption and the generation of gases such as H_2_S and NH_3_ in hydrodesulfurization and hydrodenitrogenation, respectively, or hydrogen evolution in the HER. Thus, it is assumed that the active catalyst sites for hydrotreating are also active for the HER. For example, it was seen that metal sulfides and phosphides, which have already been applied in hydrotreating, also showed a notable performance in the HER. Thus, searching for new ideas and active materials in hydrotreating catalysts can lead to the fabrication of efficient HER electrocatalysts.

#### 6.1.2. Taking advantage of density functional theory

Investigating the electronic structure of materials with quantum mechanics is an excellent, mechanistic, and accurate way to understand their nature. However, the vast number of equations and their complexity in many-particle problems make the use of quantum mechanics difficult. For example, investigating a nanocluster with 100 members of Ni_2_P and Ni_3_S_2_ with quantum mechanics and the Schrödinger equation leads to 21 300- and 34 800-dimensional problems, respectively. However, density functional theory (DFT) as a powerful tool can make these calculations simpler. Investigating electrocatalysts in terms of hydrogen adsorption energy, activity, thermodynamic stability of their different crystalline planes, the effect of different dopants, and many other aspects by DFT gives a much better and clearer vision for future works, making use of all their advantages.

#### 6.1.3. Synthesis of multi-constituent composites

It was elaborated in Section 5 that in composites, each material or group of materials has an effect on the properties of the base material. Thus, it is expected that the use of multi-constituent composites composed of different materials whose properties have been modified from different aspects can be a viable solution for the efficient electrocatalysis of the HER, as was demonstrated in the works by Pan *et al.*^125^ and Chen *et al.*^126^ In addition, combining different materials may have synergic effects, where a composite may perform even better than expected. Of course, this idea needs optimization of type and amount of combined materials, which can be a good area to focus on in future studies.

#### 6.1.4. Unsteady performance/dissolution of electrocatalysts

A steady-state performance is one of the most important properties of an electrocatalyst for industrial applications. However, nickel sulfide and phosphide electrocatalysts usually have an unsteady performance. The dissolution of electrocatalysts in electrolytes is one of the main reasons for their unsteady performance. In addition to causing unsteady-state performances, the dissolution of electrocatalysts in applications such as the chlor-alkali process, in which the electrolyte (NaOH) is the main product of the process, contaminates the electrolyte. This matter has been discussed in several works.^15,16,70,102^ For example, Ma *et al.*^102^ reported that the sulfur content of an NiS_2_ electrocatalyst decreased significantly from 45.1% to 1.5% during 3 h of polarization in 1.0 M KOH solution or in another work, Laursen *et al.*^70^ reported that about 50% of the nickel content of the Ni_2_P pellet was dissolved in solution after 20 h of electrolysis in 1.0 M H_2_SO_4_. Thus, some strategies should be considered to synthesize and fabricate robust electrocatalysts to stabilize their performance and prevent their dissolution.

### 6.2. Nickel sulfides

#### 6.2.1. High onset potential

The onset potential is one of the essential properties of electrocatalysts that determine their behavior in higher overpotentials. However, nickel sulfide electrocatalysts mostly have a high onset potential, increasing the overpotential at higher current densities. Although the onset potential is an intrinsic property, it can be improved by combining nickel sulfides with proper materials. One reason for the high onset potential of electrocatalysts is their poor conductivity and electron transfer ability. Thus, combining nickel sulfides with conductive materials can be a potential solution to overcome these issues. In this regard, carbonaceous materials have shown great potential to decrease the onset potential of other materials such as nickel phosphides.^77,78^ Thus, adding these materials to the structure of nickel sulfides can be considered a potential solution that should be examined in future works.

#### 6.2.2. Most active materials: need for further studies

As previously mentioned, Ni_3_S_2_ seems to be the most active phase of nickel sulfides in alkaline media. Although some studies have reported this finding, it needs to be further confirmed by more works. In addition, different crystalline phases of nickel sulfides rarely have been compared in acidic media, and the available findings in this regard need to be further confirmed by more studies. Thus, studying the phase-dependence performance of nickel sulfide electrocatalysts in both acidic and alkaline media can be considered one of the future topics in HER electrocatalysis using nickel sulfides.

#### 6.2.3. Role of sulfur in nickel sulfide electrocatalysts

Although several articles have been published on the application of nickel sulfide electrocatalysts in the HER, the role of sulfur in their performance is still unknown thus far. Accordingly, by determining the role of sulfur, better approaches for tuning nickel sulfides can be developed. Thus, a mechanistic study must clarify and uncover this issue.

#### 6.2.4. Various phases of nickel sulfides

According to the nickel–sulfur phase diagram, there are at least 6 distinct crystalline phases of nickel sulfide, including Ni_3_S_2_, Ni_7_S_6_, Ni_9_S_8_, NiS, Ni_3_S_4_, and NiS_2_, where each of them has unique properties.^127^ However, only Ni_3_S_2_, NiS, and NiS_2_ have been widely applied in the HER, while the other crystalline phases have been rarely investigated.^63,84^ Thus, there may be good opportunities to find better HER performances in the less studied crystalline phases.

#### 6.2.5. Preferential growth

The different planes of a crystalline structure have very different properties. This matter has been revealed or predicted in many studies. For example, Wang *et al.*^62^ investigated the activity of the different planes and active sites of NiS with DFT calculation and experiment integration. They showed that one sulfur site in the (111) plane and one nickel site and two sulfur sites in the (113) plane of NiS have activity comparable to the well-known (111) plane of Pt. This and many other examples indicate that synthesizing materials with the preferential growth of specific planes can be a good approach to prepare more active electrocatalysts.

### 6.3. Nickel phosphides

#### 6.3.1. Developing facile synthesis methods

Nickel phosphides usually are synthesized through thermal decomposition or VSR. However, although these methods have some advantages, there are several HSE problems associated with them, ranging from the use of toxic solvents to releasing PH_3_, which is a dangerous and poisoning gas. Also, they operate at high temperatures, which is not preferable. Moreover, simple synthesis methods such as the solvothermal method have limitations for the synthesis of P-rich crystalline phases and nickel phosphides that are more phosphorus-rich than Ni_2_P, *i.e.*, phases ranging from Ni_5_P_4_ to NiP_3_ have not been synthesized through this method to date. In addition, although the electrodeposition method can be employed to synthesize crystalline phases of nickel sulfide, no report was found regarding the direct electrodeposition synthesis of crystalline phases of nickel phosphides. Thus, there is a need for the development of facile synthesis methods with lower temperature and higher safety and the ability to synthesize various nickel phosphides.

#### 6.3.2. Looking for better performance in P-rich nickel phosphides

As demonstrated in Section 5, the activity of nickel phosphides in the HER increases preparing more P-rich phases. Phosphorus-rich nickel phosphide electrocatalysts also show higher stability than nickel-rich nickel phosphides. Thus, if it is desired to achieve a better performance in the HER by using nickel phosphide electrocatalysts, P-rich nickel phosphides will be more suitable. To the best of our knowledge, the most P-rich crystalline phase of nickel phosphides, *e.g.*, NiP_3_, has not been studied in the HER, and thus there is an excellent opportunity in this regard. Also, there is a wide range of ideas for fabricating new electrocatalysts by creating different alloys, composites, and dopants to tune and improve the performance of the pristine P-rich nickel phosphides, which can be considered a good topic of study.

#### 6.3.3. Mechanistic study of synthesis methods

In Section 4, some hypotheses were proposed about the synthesis of nickel phosphides, such as a higher temperature and longer synthesis time in the solvothermal method favor the formation of Ni_12_P_5_ compared to Ni_2_P and P-rich nickel phosphides cannot be synthesized through the solvothermal method. Although these claims have been observed in several studies, they need further confirmation in future works. Also, it can be helpful if a mechanistic study using DFT or thermodynamics calculations can uncover the reasons behind these observations. In addition, the affecting parameters of the electrodeposition method in synthesizing nickel phosphide electrocatalysts have not been well studied. For example, as was pointed out in Section 4.1, there are still inconsistent data about how temperature affects the phosphorus content of the synthesized coating. Therefore, it is necessary to study the effect of these parameters using a reliable and accurate manner.

## Author contributions

Ali Shahroudi: conceptualization, data curation, investigation, project administration, supervision, visualization, writing original draft, writing review & editing. Mahsa Esfandiari: data curation, investigation, writing original draft. Sajjad Habibzadeh: conceptualization, project administration, supervision, writing review & editing.

## Conflicts of interest

There are no conflicts to declare.

## Supplementary Material
